# Illuminati: a form of gene expression plasticity in *Drosophila* neural stem cells

**DOI:** 10.1242/dev.200808

**Published:** 2022-11-18

**Authors:** Alix Goupil, Jan Peter Heinen, Riham Salame, Fabrizio Rossi, Jose Reina, Carole Pennetier, Anthony Simon, Patricia Skorski, Anxela Louzao, Allison J. Bardin, Renata Basto, Cayetano Gonzalez

**Affiliations:** ^1^ Institut Curie, Paris Science et Lettres Research University, Centre National de la Recherche Scientifique, Unité Mixte de Recherche UMR144, Biology of Centrosomes and Genetic Instability Laboratory, 75005 Paris, France; ^2^ Institute for Research in Biomedicine (IRB Barcelona), Cell Division Laboratory, Cancer Science Programme, The Barcelona Institute of Science and Technology, Baldiri Reixac, 10, 08028 Barcelona, Spain; ^3^ Institut Curie, PSL Research University, CNRS UMR 3215, INSERM U934, Stem Cells and Tissue Homeostasis Group, 75005 Paris, France; ^4^ Catalan Institution for Research and Advanced Studies (ICREA), 08010 Barcelona, Spain

**Keywords:** *Drosophila*, Fluorescent reporters, Genome instability

## Abstract

While testing for genome instability in *Drosophila* as reported by unscheduled upregulation of *UAS-GFP* in cells that co-express *GAL80* and *GAL4*, we noticed that, as expected, background levels were low in most developing tissues. However, GFP-positive clones were frequent in the larval brain. Most of these clones originated from central brain neural stem cells. Using imaging-based approaches and genome sequencing, we show that these unscheduled clones do not result from chromosome loss or mutations in *GAL80.* We have named this phenomenon ‘Illuminati’. Illuminati is strongly enhanced in *brat* tumors and is also sensitive to environmental conditions such as food content and temperature. Illuminati is suppressed by *Su(var)2-10*, but it is not significantly affected by several modifiers of position effect variegation or Gal4::UAS variegation. We conclude that Illuminati identifies a previously unknown type of functional instability that may have important implications in development and disease.

## Teaser

**Summary:** ‘Illuminati’ is a type of functional instability, caused by epigenetic mechanisms, that occurs most frequently in larval neuroblasts and is enhanced in a neuroblast-derived tumor in *Drosophila*.

## INTRODUCTION

**Fig. 1.**
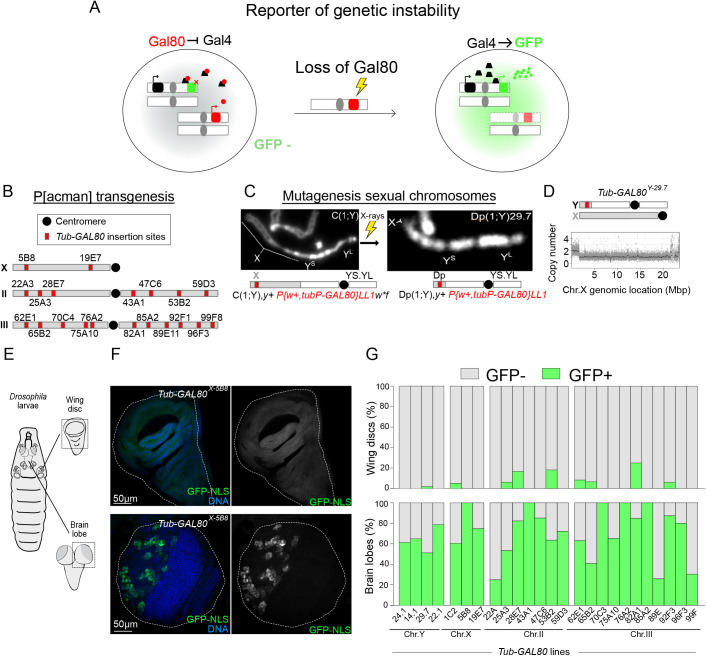
**A novel strategy to monitor genetic instability based on the Gal80::Gal4::UAS system.** (A) Schematic representation of the genetic system to monitor GI in any *Drosophila* tissue. Cells that carry a *UAS-GFP* transgene and express *GAL4* cannot express GFP in the presence of the repressor protein Gal80. GI affecting Gal80 function in these cells results in GFP-expressing clones. (B) Representative map of the *Tub-GAL80* insertion sites on *Drosophila* chromosomes X, II and III obtained by P[acman] transgenesis. (C) Strategy to generate a duplication of the distal part of the X chromosome to the Y chromosome carrying a *Tub-GAL80* insertion using recombination followed by X-ray mutagenesis. Chromosomes are labeled with DAPI. (D) Graph showing X chromosome copy number derived from sequencing of *Tub-GAL80^Y-29.7^* males. X and Y chromosomes are schematized above the graph: the *Tub-GAL80* cassette (red square) is located on a small part of the X chromosome (gray) duplicated to the Y chromosome (white). (E,F) Schematic representation of the *Drosophila* L3 larvae (E) and images (F) of wing disc (top) and brain lobe (BL) (bottom) for *Tub-GAL80^X-5B8^* line. Whole-mount tissues were labeled for GFP (green and gray) and with DAPI for DNA (blue). White dotted lines delimitate tissues. (G) Bar graphs summarizing the percentage of wing discs (top) and brain lobes (bottom) without (gray) and with (green) GFP signal from all *Drosophila* lines carrying one copy of the *Tub-GAL80* cassette on the indicated chromosomes (*n*=7-20 wing discs/condition and *n*=14-88 BLs/condition).

Genetic instability (GI), which is used here as a general term to describe both variations in chromosome number and structural alterations in DNA, is known to be linked to a variety of diseases including developmental disorders and cancer. Whole-genome sequencing (WGS) and single-cell analysis have been ground-breaking techniques to identify the frequency and type of GI in health and disease (Knouse et al., 2014; Lee et al., 2016). However, single-cell WGS is cumbersome and expensive, and is not well suited to provide time-resolved data. Building on previous work (Carmena et al., 1991; Siudeja et al., 2015; Szabad and Würgler, 1987; Szabad et al., 2012), we have conceived a fluorescence-based method that circumvents these limitations. This method is based on the tripartite Gal80::Gal4::UAS system. GI affecting the function of the repressor Gal80 in cells that carry a *UAS-GFP* transgene and co-express *GAL80* and *GAL4* generates an irreversible upregulation of GFP expression, which labels the cell in which the GI event took place as well as its offspring (Fig. 1A). The frequency and size of such GFP clones provide quantitated estimates of the extent of GI and the time in development when the triggering GI event occurred. Indeed, these reporters are sensitive to all GI types (e.g. chromosomal instability, copy number variations, point mutations, etc.) and can be used to study somatic mosaicism *in vivo* at a one-cell, one-GI-event resolution. Analysis of 25 *GAL80* fly lines carrying these reporters in different locations in chromosomes X, Y, II and III revealed that GFP^+^ cells are rare in most tissues, which is consistent with the expected low levels of GI under normal conditions. Strikingly, however, GFP^+^ cells were relatively abundant in larval brains in which most GFP^+^ clones contained one neural stem cell and its progeny. Using a variety of methods, we show that, unexpectedly, these GFP^+^ cells do not result from chromosome loss or mutations in *GAL80*. Instead, they seem to result from a new type of gene expression instability that we have named Illuminati.

## RESULTS

### A novel strategy to monitor genetic instability based on the Gal80::Gal4::UAS system

To generate tools to determine the background level of GI in different cell lineages during *Drosophila* development, we made use of the repressive effect of Gal80 on the Gal4::UAS transcription activation system (Brand and Perrimon, 1993; Pfeiffer et al., 2010). Flies that carry a *UAS-GFP* transgene and express both *GAL4* and *GAL80* from constitutive promoters cannot express GFP because Gal80 prevents UAS-bound Gal4 from interacting with the transcriptional machinery (Ma and Ptashne, 1987). GI affecting Gal80 function will therefore switch on GFP expression, resulting in a clone of green fluorescent cells that will include the cell of origin and its offspring. Such reporters could be used to quantify and to time the onset of GI in any tissue (Fig. 1A).

We generated a collection of *Drosophila* strains carrying *GAL80* insertions in all four major chromosomes (X, Y, II and III). To generate the lines carrying *GAL80* on the X, II and III chromosomes, we designed a new vector carrying a version of *GAL80* optimized for *Drosophila* codon usage (Pfeiffer et al., 2010) under the control of the ubiquitous tubulin 1α promotor (*Tub-GAL80*) (Lee and Luo, 1999; O'Donnell et al., 1994). To minimize the risk of undesired positional effects that could affect *GAL80* expression, we generated these lines using the targeted insertion φC31-recombination attB P[acman] system (Venken et al., 2006). A total of 20 *Drosophila* transgenic lines, each carrying one copy of the *Tub-GAL80* transgene inserted at different genomic regions, was obtained (Fig. 1B). Each of these lines is referred to as *Tub-GAL80* followed by the designation of the chromosome and insertion site in superscript (e.g. *Tub-GAL80^X-5B8^* stands for *GAL80* inserted on the X chromosome at location 5B8).

To generate *GAL80* insertions on the Y chromosome, we first recombined the *P{w+, tubP-GAL80}LL1* transgene *Tub-GAL80^X-1C2^* located distally on the X chromosome (Lee and Luo, 1999) into a *C(1;Y)* chromosome that carries a fully functional fusion between the X and Y chromosomes sharing a single centromere. The resulting *C(1;Y) P{w+, tubP-GAL80}LL1* was then subjected to X-ray mutagenesis to generate large deletions that remove most of the X chromosome, leaving only the most distal part of the X chromosome of *C(1;Y)* attached to a fully functional Y chromosome; i.e. transforming the original *C(1;Y) P{w+, tubP-Gal80}LL1* in a *Dp(1;Y) P{w+, tubP-GAL80}LL1* (Cook et al., 2010). From a total of 38,206 *C(1;Y) P{w+, tubP-GAL80}LL1* chromosomes, we obtained four different lines, which will be referred to as *Tub-GAL80^Y^* followed by a number to identify each line. Banding of mitotic chromosomes labeled with DAPI revealed the corresponding *Dp(1;Y)* as a short euchromatic region attached distally to the Y heterochromatin (Fig. 1C). WGS identified the exact break point of these duplications that ranged in size from 1.08 Mbp to 1.66 Mbp (Fig. 1D).

To determine the suitability of our collection of GI reporters, we quantified the basal rate of GFP expression in larval imaginal discs and brains (Fig. 1E). As expected, regardless of the chromosome where the *GAL80* transgene was inserted (X, Y, II or III), we found no GFP-expressing cells in the majority of wing discs (416/429 in total) (Fig. 1F,G; compare with Fig. S1A for controls and Fig. S1D). In ten out of the 13 wing discs that contained GFP^+^ cells, GFP expression was restricted to only a few cells within the whole GFP-expressing tissue (ten wing discs from seven different *Tub-GAL80* constructs). Interestingly, only one single *GAL80* line- (*Tub-GAL80^III-82A1^*) had a high number of GFP^+^ cells, and this only occurred in three out of 12 wing discs analyzed (Fig. S1C,D). In stark contrast to the discs, most larval brains examined presented clusters of GFP^+^ cells at rates that varied substantially, but were always at least one order of magnitude greater than that observed in wing discs (Fig. 1F,G; compare with Fig. S1B for controls, Fig. S1E).

To determine whether the observed differences in GFP clone frequency between wing discs and brains might be explained by differences in cell number and mitotic activity between the two tissues, we quantified mosaic analysis with a repressible cell marker (MARCM) [heat-inducible recombination at flippase recognition target (FRT) sites by flippase (FLP)] clones (Fig. S2A). We only observed very minor differences in the frequency of GFP^+^ cell clusters in wing discs (mean=22.4, *n*=7) and brain lobes (mean=16.6, *n*=10) (Fig. S2B-D). Thus, differences in cell number and mitotic activity cannot account for the high frequency of GFP^+^ cells in the brain compared to that in wing discs.

Our results show that, as expected, the majority of the *Tub-GAL80* insertions generated in this study efficiently repress *GAL4::UAS-GFP* expression in wing discs. They also confirm that GI is a very rare event in wing disc cells. Our results also reveal a relatively high number of unexpected GFP^+^ cells in the larval brain, which we decided to analyze in more detail.

### The majority of unexpected GFP^+^ clones represent central brain neuroblasts and their lineage

**Fig. 2.**
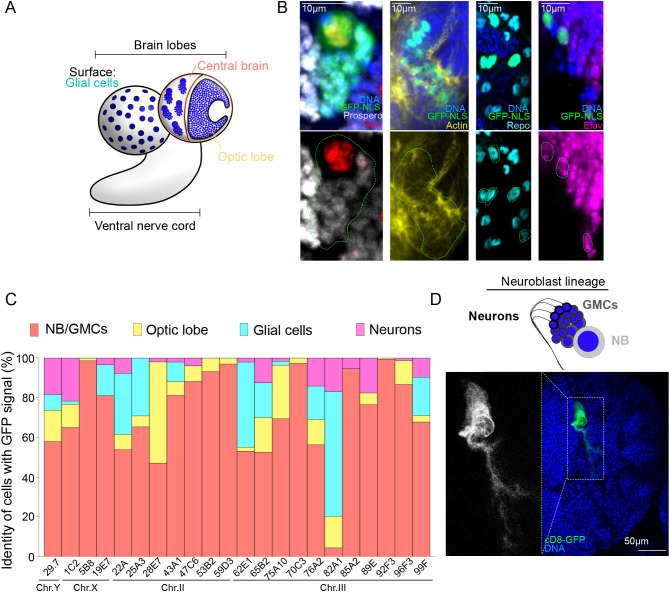
**The majority of GFP-positive clusters correspond to NB lineages in larval brains.** (A) Schematic representation of the *Drosophila* larval brain and its different cell types. (B) Representative images of whole-mount brain lobes labeled for GFP (green) and for specific markers of different cell types: Dpn^+^ NBs (red) with Prospero^weak^ GMCs (gray); cells of the NE from the optic lobes, distinguishable by the specific F-actin organization (yellow); Repo^+^ glial cells (cyan); and individual Elav^+^ neurons (pink). DNA is shown in blue. Green dotted lines surround GFP^+^ clusters or cells. (C) Bar graph showing the percentage of cells showing GFP^+^ signals for each cell type of the brain lobe: NB lineage (orange), optic lobe cells (yellow), individual glial cells (cyan) and individual neurons (pink) (*n*=7-43 BLs/condition). (D) Representative image of a whole-mount brain and magnified view of a GFP^+^ cluster containing the full lineage of a single NB, including neurons, ganglion cells, GMCs, and the NB itself from a larva expressing the membrane marker *UAS-cD8-GFP* in addition to *GAL4* and *Tub-GAL80^Y-29.7^*.

The larval brain lobe (BL) can be divided into two main regions, the central brain and the optic lobe (Fig. 2A). The central brain is composed of neural stem cells, also called neuroblasts (NBs), which divide asymmetrically to self-renew and give rise to smaller and more committed progenitors, the ganglion mother cells (GMCs) (Bello et al., 2008; Boone and Doe, 2008; Homem and Knoblich, 2012). The optic lobe consists of two proliferative centers, the inner and the outer, which correspond to a pseudo-stratified epithelium called the neuroepithelium (NE). NE cells give rise to medulla NBs that generate neurons and glia necessary for the development of the visual system of the fly. Further, perineural and sub-perineural glial cells with large nuclei are found at the superficial layer of the brain (Pereanu et al., 2005). All these cell types are easily distinguishable by their morphology, position in the brain and expression markers. For instance, the signals Dpn^+^, Dpn^−^/Propero^weak^, Elav^+^ and Repo^+^ indicate NBs, GMCs, neurons and glial cells, respectively. The NE is distinguished by the morphology of its constituent cells revealed by actin (Rujano et al., 2013).

Taking advantage of this wealth of markers, we determined the identity of the GFP^+^ cells in *GAL80::GAL4::UAS-GFP* brains. Analysis of 590 brain lobes from all 22 fly lines containing *Tub-GAL80* insertions in the X, Y, II and III chromosomes (a minimum of 14 brain lobes per *Tub-GAL80* insertion line) revealed that the number and identity of GFP^+^ cells varied between different *Tub-GAL80* lines (Fig. 2B,C). Three extreme examples were *Tub-GAL80^X-5B8^*, which presented a high number of GFP^+^ cells (most of which were NBs and associated GMCs), very few cells in the optic lobe, and no neurons or glial cells; *Tub-GAL80^III-82A1^*, which also showed a high number of GFP^+^ cells, mostly neurons and glia and few NBs/GMCs clusters; and *Tub-GAL80^II-22A^*, in which GFP^+^ cells were rare. However, notwithstanding inter-line variability, plotting the mean frequency for each cell type for all the *Tub-GAL80* lines showed that most GFP^+^ cells were located in the central brain and most of them represented NBs and associated GMCs (Fig. 2C). Using membrane-bound *CD8::GFP* as a reporter, we frequently found GFP^+^ clusters that contained one NB and its lineage including neurons (Fig. 2D). Importantly, we found that MARCM clones were more frequent in the optic lobe (58%) than in the central brain (13%) (Fig. S2E), hence discarding the possibility that differences in mitotic activity caused the predominance of the unexpected GFP^+^ cells in the central brain. We did not find a trend in the position of GFP^+^ cells among the many brain lobes analyzed.

As the majority of GFP^+^ clones observed in the larval brain originated from NBs, which are neural stem cells, we wondered whether similar clones were also present in tissues generated by other types of stem cells (SCs). To answer this question, we analyzed testes and ovaries, which contain several types of both somatic and germline SCs. We found no GFP-expressing cells in any of the samples that we analyzed, which included 129 adult testes from *Tub-GAL80^Y-29.7^* males, and 96 egg chambers and 50 larval ovaries from *Tub-GAL80^X-1C2^* and *Tub-GAL80^X-5B8^* females, respectively (Fig. S2F,G).

Taken together, these results demonstrate that although the frequency and cell types do vary among different *GAL80* lines, most GFP^+^ cells are present in the larval brain and correspond to central brain NBs and their lineage. They also show that the unexpected generation of GFP-expressing cells does not appear to be a general feature of SCs, certainly not at the rate that it occurs in central brain NBs.

### GI does not account for the unexpected appearance of GFP^+^ cells in the brain

Two mechanistically different types of GI could account for the appearance of GFP^+^ cells in the brain: aneuploidy, i.e. loss of the chromosome that carries the *GAL80* transgene, or mutations in the *GAL80* gene, leading to lack of expression or to expression of inactive mutant forms of Gal80.

**Fig. 3.**
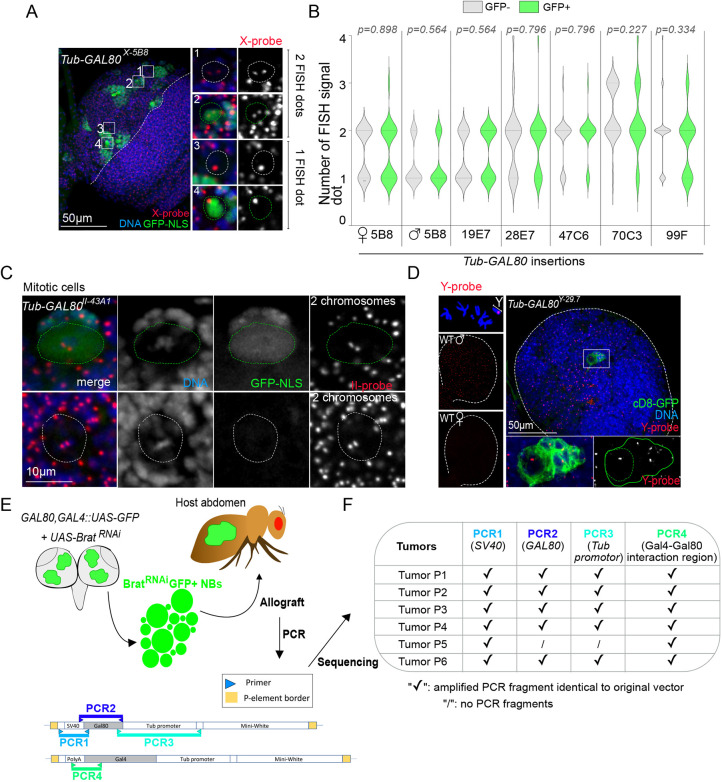
**GFP-positive cells are not caused by aneuploidy or GI in the larval brain.** (A,C,D) FISH of whole-mount brains using probes for chromosomes X (A), II (C) and Y (D) (red and gray) combined with GFP labeling (gray and/or green) and DAPI for DNA (gray and/or blue). White dotted lines surround brain lobes and/or GFP^−^ NBs. Green continuous and dotted lines surround GFP^+^ clusters and cells, respectively. (B) Violin plot representing the number of FISH signal dots in GFP^+^ and GFP^−^ cells. FISH signals correspond to the chromosomes X, II or III for conditions in which *Tub-GAL80* was inserted at positions 5B8 (*n*=43 cells for females and *n*=50 cells for males) and 19E7 (*n*=47 cells), 28E7 (*n*=50 cells) and 47C6 (*n*=50 cells), and 70C3 (*n*=47 cells) and 99F (*n*=60 cells), respectively. FISH signals are variable between conditions but similar between GFP^+^ (green) and GFP^−^ (gray) cells from the same condition. Statistical significance was determined by a Mann–Whitney test and the *P*-values are indicated. (C) GFP^+^ and GFP^−^ NBs are diploid as they present two dots for the two chromosomes II. (D) FISH using a probe for the Y chromosome-specific satellite AATAC. In metaphase cells from squashed preparations, the fluorescence signal (red) maps to the long arm of the Y chromosome (top left). In whole-mount control larval brains, there is one distinct fluorescent dot per cell in male and no signal at all in female brains. All (*n*=33) GFP clones analyzed from *Tub-GAL80^Y-29.7^* larval brains presented a Y chromosome-specific FISH signal. (E) Schematic representation of the protocol used to sequence the *GAL80* and *GAL4* genes in GFP^+^ brain cells. *UAS-brat^RNAi^* was used to induce tumors and, thus, drastically increased the GFP^+^ population to obtain sufficient DNA. *Tub-GAL80* coding and regulatory sequences were amplified by PCR and subsequently sequenced. (F) Table summarizing the results obtained for each tumor line (*n*=6 tumor samples).

To investigate whether GFP^+^ cells were aneuploid, we performed fluorescence *in situ* hybridization (FISH) using probes generated against the X, II and III chromosomes in brains containing GFP^+^ cells. In interphase cells, precise ploidy quantification is not simple because the number of FISH-positive dots can range from one to four (for diploid cells), depending on both cell-cycle stage and chromosome pairing (Joyce et al., 2012). However, we found that FISH signals were similar between GFP^+^ and GFP^−^ cells (*n*=347 cells) (Fig. 3A,B). Moreover, FISH in mitotic NBs, in which FISH signals can be assigned to individual chromosomes (Carmena et al., 1993; Gatti et al., 1994), confirmed that GFP^+^ cells were not aneuploid (Fig. 3C). To unequivocally test whether GFP^+^ cells resulted from chromosome loss, we used a probe against the Y-specific satellite AATAC (Fig. 3D) (Bonaccorsi and Lohe, 1991) in *Tub-GAL80^Y-29.1^* brains. We found that all GFP^+^ clones (*n*=33) presented a Y-specific FISH signal that could not be distinguished from that of the neighboring cells that did not express GFP. These results show that the high frequency of GFP^+^ cells observed in our collection of *Tub-GAL80* lines cannot be explained by the loss of the *GAL80*-bearing chromosome.

We then decided to sequence the *Tub-GAL80* transgene to ascertain whether mutations could account for loss of Gal80 function. This is technically challenging because GFP^+^ cells are orders of magnitude less abundant than the surrounding cells that do not express GFP. To circumvent this problem, we generated flies that, in addition to the usual combination of *GAL80::GAL4::UAS-GFP* transgenes, carried a fourth transgene encoding *UAS-brat^RNAi^*. We reasoned that if the loss of Gal80 function, which leads to transcription of the *UAS-GFP* transgene, leads to the concomitant transcription of *UAS-brat^RNAi^*, certain GFP^+^ cells could develop as *brat* tumors that could be cultured in allografts. This in principle would make it possible to isolate large quantities of DNA from GFP^+^ cells (Fig. 3E). Using this approach, we obtained six tumors from which we sequenced the corresponding *Tub-GAL80* regulatory and coding sequences. We also amplified and sequenced the region encoding the C-terminal Gal80-binding site of Gal4 because mutations or deletions in this region result in constitutively active Gal4 in the presence of Gal80 (Ma and Ptashne, 1987). We found that in five out of six tumor samples, the *Tub* promoter, *GAL80* coding sequence and *SV40* terminator sequence (i.e. PCR3, PCR2 and PCR1, respectively, in Fig. 3F) were identical to those contained in the original transgene *P{w^+^, tubP-GAL80}LL1*. In the remaining sample, PCR failed to amplify fragments PCR2 and PCR3. In all six tumor samples, the sequence corresponding to the Gal80-binding site of Gal4 was found to be wild type (Fig. 3F).

These results strongly suggest that the majority of GFP^+^ cells observed in *GAL80::GAL4::UAS-GFP* larval brains represent a type of functional instability that is caused neither by mutation nor by chromosome loss. We will henceforth refer to this unknown phenomenon of ‘illumination’ of NBs in the brain as Illuminati.

### Analysis of *Tub-GAL80* lines alerts its use for MARCM analysis

MARCM clones are widely used by the *Drosophila* community to control gene expression and as a method for lineage tracing (Lee and Luo, 1999; Ren et al., 2016). MARCM is based on FLP-driven mitotic recombination at specific FRT sites. In *Tub-GAL80/m* transheterozygous cells (where *m* represents a mutant of interest), MARCM generates a pair of clones. One of the clones, which is labeled by *UAS-GFP* expression, lacks *Tub-GAL80* (i.e. *GAL80* loss of heterozygosity or LOH) and is homozygous for the mutant in question. The twin clone, which remains GFP^−^, is homozygous for *Tub-GAL80* and lacks the mutant of interest (Fig. S3A).

As far as larval brains are concerned, our results predict that a fraction of MARCM GFP^+^ cells might not result from *GAL80* LOH. To test this possibility, we induced MARCM *sas4* mutant (*sas4^mut^*) clones by crossing *heat-shock*-*FLP; FRT82B sas4^mut^* flies with *UAS-GFP-NLS; Tub-GAL4, FRT82B Tub-GAL80* from the Bloomington *Drosophila* Stock Center (BDSC, #5132) (Lee and Luo, 1999). The *sas4* gene encodes a protein essential for centriole duplication and *sas4^mut^* cells lack centrosomes (Basto et al., 2006). Following a 1 h heat shock at 37°C to induce FLP-mediated recombination, we found that most GFP^+^ clones (78.3%, 72/92, 22 BLs) had no centrosomes as revealed by immunofluorescence against Sas4 and Cnn (Fig. S3B). However, the remaining GFP^+^ clones (21.7%, 20/92, 22 BLs) did contain centrosomes (Fig. S3C). Moreover, GFP^+^ clones were also observed in control brains that were not subjected to heat-shock treatment (33 GFP^+^ clones in 19 BLs, 1.7±1.6 GFP^+^ clones/BL, indicated as mean±s.d.) (Fig. S3E,F). Importantly, in contrast to brains, wing discs presented GFP^+^ clones only after heat shock (31 GFP^+^ clones in seven discs) and all of them lacked centrosomes (Fig. S3D,G). These results show that a significant fraction of the clones observed in MARCM experiments in the larval brain are caused by Illuminati and, therefore, are not homozygous for the mutant of interest, as intended.

### Stoichiometry imbalance contributes to Illuminati

**Fig. 4.**
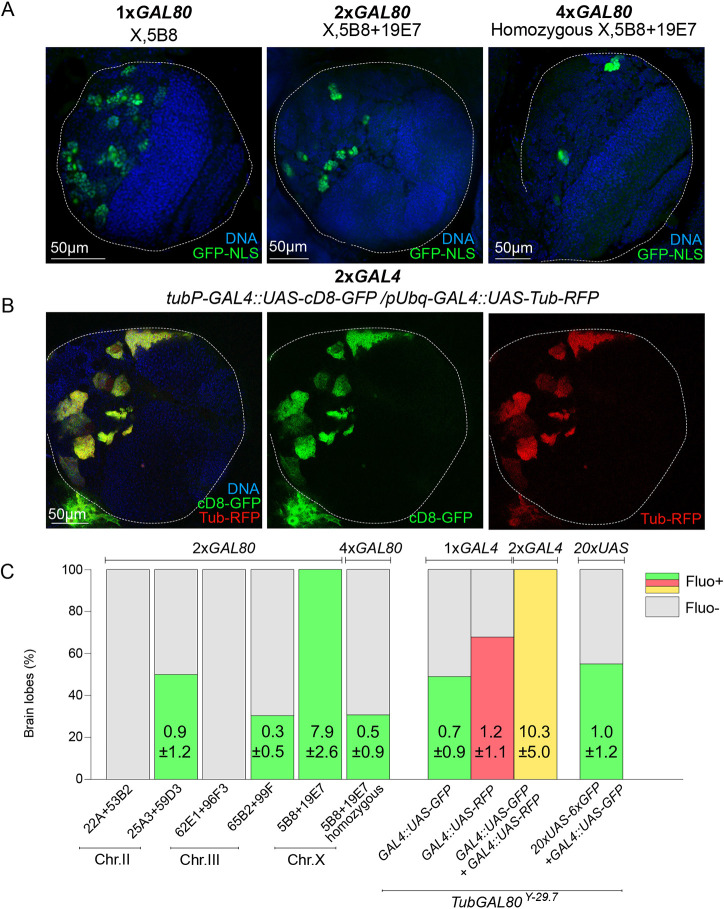
**Gal4/Gal80 stoichiometry contributes to the presence of Illuminati cells in the larval brain.** (A,B) Images of whole-mount brain lobes showing DNA (blue), GFP labeling (green) (A,B) and RFP labeling (red) (B). White dotted lines delimitate brain lobes. (C) Bar graph showing the percentage of brain lobes without (gray) and with fluorescence signals (green, red or yellow). The numbers represent average±s.d. of GFP^+^ cells in each condition (*n*=8-51 BLs/condition). To compare with individual insertions, see Fig. S1E.

To get further insight into the molecular mechanism of Illuminati, we studied the consequences of changing the 1:1:1 ratio of *GAL80::GAL4::UAS*. We first determined the effect of increasing the number of *Tub-GAL80* transgenes. To this end, we generated five different *Drosophila* recombinant lines harboring two copies of *Tub-GAL80* each inserted at distant loci in the same chromosome (one line for the X chromosome and two lines each for chromosomes II and III), referred to as *2×Tub-GAL80*. We found that Illuminati was fully suppressed in all brain lobes from two lines, whereas in the remaining three lines, Illuminati cells (i.e. GFP^+^ cells) were still detected (Fig. 4A,C), albeit less frequently. One particularly interesting case was line *GAL80^X-5B8,19E7^*. As shown in Fig. 4C, all *GAL80^X-5B8,19E7^*/X heterozygous brains presented GFP^+^ cells at a rate of nearly eight GFP^+^ clones per brain. In contrast, in *GAL80^X-5B8,19E7^*/*GAL80^X-5B8,19E7^* homozygous larvae, only a third of the brains were GFP^+^ and the frequency of Illuminati clones per brain lobe dropped to 0.5.

Consistent with the inhibitory effect of several *GAL80* transgenes, Illuminati was notably enhanced in flies that carried two *GAL4* transgenes (Fig. 4B,C). This increase in Illuminati frequency was largely accounted for by clones in the central brain (clone frequency was not increased in the optic lobe) containing NBs and associated GMCs. Interestingly, in individuals carrying two *GAL4* transgenes, the fluorescence signal was remarkably homogeneous within a given clone, but it was rather variable from clone to clone. This was also observed in lobes with a single *GAL4* transgene when the frequency of Illuminati was high. Illuminati clones in individuals carrying two *GAL4* transgenes together with *UAS-GFP* and *UAS-RFP* co-expressed both fluorescent proteins at levels with roughly similar fluorescence intensities (i.e. clones that presented weak or strong GFP signals also presented weak or strong RFP signals, respectively) (Fig. 4B). The presence of one additional UAS transgene that carried a tandem repeat of 20× UAS sequences had no effect on Illuminati frequency (Fig. 4C).

Taken together, these data are consistent with a model in which Illuminati cells arise because of Gal80 levels stochastically falling below the critical threshold that is required to efficiently suppress Gal4::UAS driven transcription. Different fluorescence intensities may reflect a dynamic range of the levels of Gal80 function, all below the threshold but still able to partially inhibit the Gal4::UAS system to a greater or lesser extent. Adding an extra *GAL4* transgene raises the threshold, thereby increasing the number of cells in which Gal80 function falls below the threshold, which indeed is reduced by an additional *GAL80* transgene.

### Illuminati NBs maintain a pattern of GFP expression that correlates with lack of *GAL80* expression

**Fig. 5.**
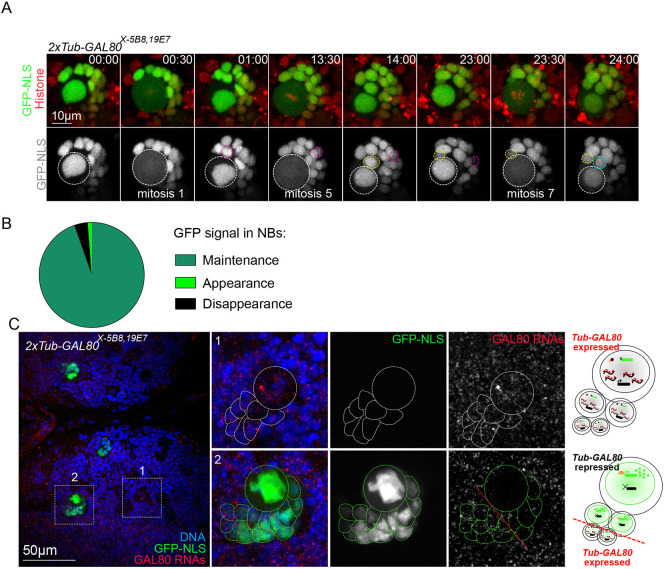
**GFP signal in Illuminati NBs is maintained throughout several consecutive divisions and correlates with lack of *GAL80* expression.** (A) Images from time-lapse movies of mitotic NBs expressing *2×Tub-GAL80^X-5B8,19E7^*, *GAL4::GFP-NLS* (green) and histone-RFP (red) to monitor GFP and chromosome dynamics. White dashed circles surround the NB. Pink, yellow and cyan circles surround the GMCs born at the first, fifth and seventh mitoses recorded in this video, respectively. (B) Pie chart of the proportion of the GFP dynamics in Illuminati NBs (*n*=167 NBs from 17 brain lobes): maintenance and clonal expansion (dark green), appearance (light green) or disappearance (black) of the GFP signal. (C) Images of whole-mount brain lobes from RNA FISH experiments with probes against the *GAL80* RNAs (red and gray in magnified views) and labeled with GFP booster (green and gray in magnified views) and DAPI for DNA (blue). Schematic representations of cells are shown next to the images. White and green dotted lines surround GFP^−^ and GFP^+^ NB/GMCs clusters, respectively. Cells on the bottom left of the red dotted line present high *GAL80* FISH signal

To obtain a dynamic view of Illuminati, we performed long-term time-lapse microscopy covering nearly two-thirds of the total proliferative window of the central nervous system during third instar larval stages. We analyzed 17 *2×Tub-GAL80^X-5B8,19E7^* brain lobes with a total number of 167 GFP^+^ events restricted to the central brain NB lineages. Regarding GFP^+^ NBs, we found that the majority (94.6%, *n*=158 out of 167) produced GFP^+^ GMCs and remained GFP^+^ through successive rounds of cell division (Fig. 5A,B). Interestingly, minor behaviors were also identified. In seven NBs (4.2%), the initial GFP signal disappeared and the NB became GFP^–^. Furthermore, *de novo* GFP appearance could only be identified in two NBs (1.2%) (Fig. 5B; Fig. S4A,B).

Regarding the major category, GFP^+^ NBs and their progeny, the intensity of green fluorescence was decreased in GMCs positioned the furthest away from the NB. This observation suggests that *GAL80* expression might have been re-established in these cells. To test this possibility, we designed FISH probes that recognize *GAL80* mRNAs. We found that GFP^+^ NBs lacked *GAL80* RNA-FISH signals that could be easily recognized in GFP^−^ cells. Notably, *GAL80* RNA-FISH signals were also detectable in the cells within the GFP^+^ NB-progeny clusters that presented low fluorescence intensities (Fig. 5C). These results show that although the probability of Illuminati taking place in a NB at any given cell cycle is relatively low, the majority of NBs retain the Illuminati status and pass on the condition to their offspring through successive cell cycles.

### Illuminati shares regulatory elements with Gal4::UAS variegation and position effect variegation, but also presents notable differences

Illuminati clones reveal an unexpected level of GFP expression variegation. There are two well-characterized types of gene expression variegation in *Drosophila*: position effect variegation (PEV) and Gal4::UAS variegation. PEV is caused by the silencing of a gene in certain cells through its proximity to heterochromatin (Elgin and Reuter, 2013). Gal4::UAS variegation, however, refers to the variegated expression of Gal4-driven UAS-genes that can be observed in *GAL4::UAS-EGFP* follicle epithelia where patches of cells exhibiting different fluorescence intensities can be distinguished (Lee et al., 2017; Skora and Spradling, 2010).

**Fig. 6.**
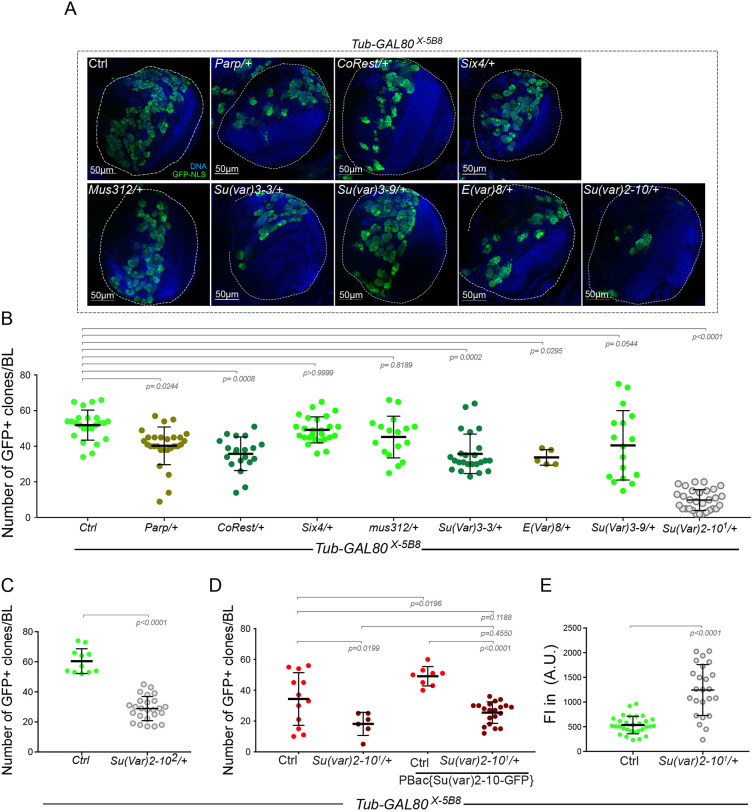
**Illuminati is influenced by certain PEV and Gal4::UAS variegation factors and strongly suppressed by *Su(var)2-10*.** (A) Images of whole-mount brain lobes showing DNA (blue) and GFP labeling (green) in the indicated genotypes. White dotted lines delimitate brain lobes. (B) Dot plot showing the number of Illuminati per brain lobe (BL) in larvae from the indicated genotypes [*n*=24 BLs in control (Ctrl), 17 BLs in *Mus312/+*, 28 BLs in *Parp/+*, 26 BLs in *Six4/+*, 24 BLs in *Su(var)3-3/+*, 30 BLs in *Su(var)2-10^1^/+*, 18 BLs in *Su(Var)3-9/+*, 6 BLs in *E(Var)8/+* and 20 BLs in *CoRest/+*]. Statistical significance was determined by a one-way ANOVA with non-parametric Kruskal-Wallis post hoc test. (C) Dot plot of the number of Illuminati/BL of *Tub-GAL80^X-5B8^* combined with *Su(var)2-10^2^/+* (*n*=11 BLs in Ctrl and 25 BLs in *Su(var)2-10^2^/+*). Statistical significance was determined by two-tailed unpaired *t*-test. (D) Dot plot of the number of Illuminati/BL in *Tub-GAL80^X-5B8^* (*n*=12) and in *Tub-GAL80^X-5B8^* combined with *Su(var)2-10^1^/+* (*n*=6), *PBac{Su(var)2-10-GFP}* (*n*=8), and *Su(var)2-10^1^/+* together with *PBac{Su(var)2-10-GFP}* (*n*=20). Statistical significance was determined by ordinary one-way ANOVA with ordinary one-way ANOVA post hoc test. (E) Dot plot representing the fluorescence intensity in arbitrary units (A.U.) of Illuminati NBs in *Tub-GAL80^X-5B8^* (*n*=30) and *Tub-GAL80^X-5B8^; Su(var)2-10^1^/+* (*n*=24). Statistical significance was determined by two-tailed unpaired *t*-test. The bars indicate the mean±s.d.

To assess the possible overlap between Illuminati and these two types of variegation, we tested the effect of selected dominant modifiers of PEV and GAL4::UAS variegation on Illuminati rates using the *Tub-GAL80^X-5B8^* line, which presents the highest frequency of GFP^+^ NBs and GMCs. The selected modifiers included: *Poly-(ADP-ribose) polymerase* (*Parp*), *Suppressor of variegation 3-3* [*Su(var)3-3*, also known as *Lsd1*], *CoRest*, *Six4*, *mutagen-sensitive 312* (*mus312*), *Enhancer of variegation 8* [*E(var)8*], *Su(var)3-9* and *Su(var)2-10* (Lee and Spradling, 2014; Schotta, 2002; Skora and Spradling, 2010; Tartof and Bremer, 1990; Tulin et al., 2002; Westphal and Reuter, 2002). *Parp* is a strong enhancer of Gal4::UAS variegation, whereas *Su(var)3-3*, *CoRest*, *Six4* and *mus312* are suppressors of Gal4::UAS variegation. In addition, *Su(var)3-3* is also a PEV suppressor. *E(var)8* is an enhancer of PEV, whereas *Su(var)3-9* and *Su(var)2-10* are suppressors. We found that the suppressors of PEV *Su(var)3-3* and *Su(var)2-10* behaved as dominant suppressors of Illuminati, as did the suppressor of Gal4::UAS variegation *CoRest*. However, other suppressors of PEV and Gal4::UAS variegation, including *Six4*, *mus*312 and *Su(var)3-9*, had no significant effect on Illuminati. Moreover, notably, *Parp* and *E(var)8*, which are enhancers of Gal4::UAS variegation and PEV, respectively, behaved as dominant suppressors of Illuminati (Fig. 6A,B). According to the DIOPT Ortholog Finder (Hu et al., 2011), *Drosophila Su(var)3-3*, *Su(var)2-10*, *CoRest* and *Parp* are orthologs of human *KDM1A*, *PIAS1*, *RCOR1*/*2* and *PARP1*, respectively, and *E(var)8* does not have a human ortholog. These results reveal that key chromatin remodeling proteins participate in Illuminati. They also reveal that Illuminati shares regulatory elements with Gal4::UAS variegation and PEV, but presents notable differences from them.

Among the mutant conditions analyzed, *Su(var)2-10^1^* was the strongest suppressor of Illuminati. We thus decided to test this condition in more detail. We first tested a different allele, *Su(var)2-10^2^* (Wustmann et al., 1989) and confirmed that it also significantly decreases the rate of Illuminati (Fig. 6C). We next tested the effect of an extra copy of wild-type *Su(var)2-10* using a fly line that carries the *PBac{Su(var)2-10-GFP}* construct that expresses a GFP-tagged wild-type *Su(var)2-10* gene. To distinguish the Su(var)2-10-GFP signal from Illuminati signals, we used a *UAS-mCD8-RFP* reporter, which labels membranes in red. We found that the *PBac{Su(var)2-10-GFP}* construct did not significantly rescue the reduction of Illuminati caused by the loss of *Su(var)2-10* in a *Tub-GAL80^X-5B8^ Su(var)2-10^2^/+* background, but significantly increased Illuminati frequency in a *Tub-GAL80^X-5B8^* background (Fig. 6D). These findings confirm *Su(var)2-10* as a positive regulator of Illuminati frequency. In addition, we noticed that the few cells affected by Illuminati in *Su(var)2-10^1^/+* larvae presented a twofold increase in mean GFP signal compared to controls (Fig. 6E), suggesting that Su(var)2-10 is also a negative regulator of Illuminati intensity.

### Investigating the sensitivity of Illuminati to stress and environmental conditions

**Fig. 7.**
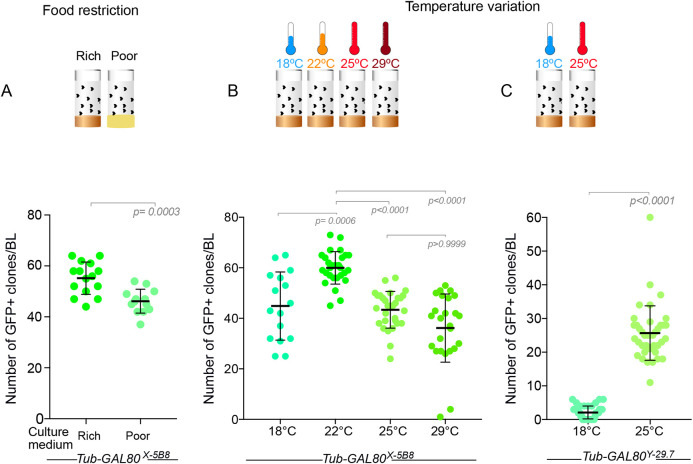
**Illuminati is influenced by environmental factors.** (A-C) Dot plot showing the number of Illuminati per brain lobe (BL) in larvae raised on different culture media (A) and at different temperatures (B,C). (A) *Tub-GAL80^X-5B8^* flies were raised on protein-rich (*n*=70 BLs from 35 brains) or -poor (*n*=62 BLs from 31 brains) medium. (B) *Tub-GAL80^X-5B8^* flies were raised at 18°C (*n*=43 BLs from 22 brains), 22°C (*n*=76 BLs from 38 brains), 25°C (*n*=34 BLs from 17 brains) or 29°C (*n*=58 BLs from 29 brains). (C) *Tub-GAL80^Y-29.7^* flies were raised at 18°C (*n*=42 BLs from 21 brains) or 25°C (*n*=39 BLs from 20 brains). Statistical significance was determined by a Mann–Whitney test. Error bars correspond to the mean±s.d.

Our results strongly suggest that Illuminati reflects a yet unidentified mechanism by which Gal80 function falls below the minimum level required for efficient inhibition of Gal4::UAS-driven transcription. If that was the case, one could expect Illuminati frequency to be sensitive to experimental conditions that impose stress and disturb gene expression. To test this hypothesis, we assessed the effect of food composition and temperature. Using the *Tub-GAL80^X-5B8^* line, which presents the highest frequency of Illuminati under normal culture conditions, we found that a protein-poor medium made of cornmeal and low yeast content significantly reduced the number of Illuminati GFP^+^ clones compared to that observed in larvae cultured in standard rich medium (Fig. 7A). In this same *Tub-GAL80^X-5B8^* line, we found that Illuminati frequency increased as the culture temperature was raised from 18°C to 22°C, and decreased at 25°C and 29°C (Fig. 7B). However, in the *Tub-GAL80^Y-29.7^* line, Illuminati frequency was strongly enhanced at higher temperatures (Fig. 7C). These results reveal that Illuminati is sensitive to environmental stimuli and that different *GAL80* insertions can respond differently to such stimuli.

### Illuminati can contribute to phenotypic instability in tumor cells

**Fig. 8.**
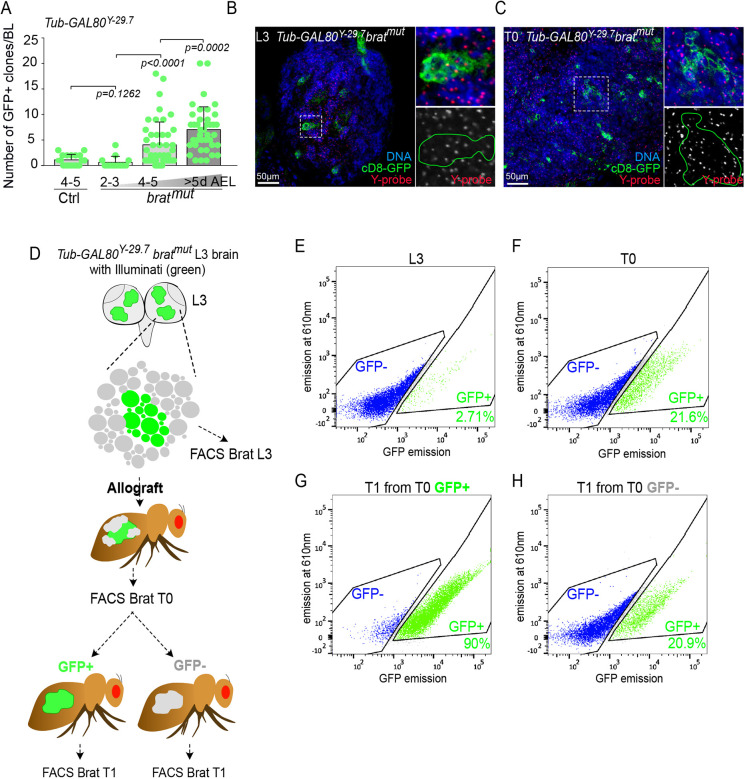
**Illuminati is maintained and can appear *de novo* in *brat*-induced tumors*.*** (A) Dot plot showing the number of Illuminati clones/brain lobe in *Tub-GAL80^Y-29.7^* control and *Tub-GAL80^Y-29.7^ brat^mut^* brains in consecutive days after egg laying (AEL). Statistical significance was determined by a Mann–Whitney test. Error bars correspond to the means±s.d. (B,C) FISH with Y chromosome probes (red and gray) of *brat^mut^* whole-mount brains (B) and tumor tissue from T0 allograft (C) combined with GFP labeling (green) and DAPI for DNA (blue). Green lines surround Illuminati clones in L3 brains (B) and T0 tumor (C) after allograft. (D) Schematic representation of the protocol to analyze Illuminati behavior and stability upon tumorigenesis based on FACS analysis and successive transplantation assays. (E-H) Representative FACS profiles of Illuminati cells. (E,F) The fraction of Illuminati cells massively increased from before (E) to after allografting (F) (*n*=2 independent experiments). (G) The rate of Illuminati cells in T1 from transplantation of T0 GFP^+^ cells is maintained. (H) A fraction of Illuminati cells emerged in T1 from transplantation of T0 GFP^−^ (*n*=1 experiment).

To determine whether Illuminati could contribute to phenotypic instability during malignant growth, we chose to study *brat* tumors. *brat* tumors originate from type II NB-lineage intermediate progenitors that are transformed into immortal NB-like tumor stem cells (Betschinger et al., 2006). To assess Illuminati frequency during *brat* tumor development, we quantified GFP clones in the *brat^K06028^* mutant (*brat^mut^*) and control flies that carried *GAL4::UAS-GFP* and *Tub-GAL80^Y-29.7^*. We chose this *GAL80* insertion on the Y chromosome because of the ease to unambiguously test for Y chromosome loss by FISH. We could not detect significant differences in Illuminati frequency between *Tub-GAL80^Y-29.7^* control and *Tub-GAL80^Y-29.7^ brat^mut^* brains before larvae reached the third instar stages (Fig. 8A). However, the rate of Illuminati cells per lobe in *brat^mut^* brains increased dramatically afterwards and remained significantly greater than in control brains, with most *Tub-GAL80^Y-29.7^ brat^mut^* brains presenting more than five Illuminati clones per brain and some presenting up to 20 clones (Fig. 8A). Importantly, FISH confirmed that all (*n*=30) GFP^+^ clones analyzed in *Tub-GAL80^Y-29.7^ brat^mut^* brains retained the Y-*GAL80* chromosome (Fig. 8B).

We then decided to follow Illuminati behavior during the period of massive growth that takes place upon allograft of larval brain tumors into adult hosts (Caussinus and Gonzalez, 2005) (Fig. 8D-F). In two independent experiments, we found that the fraction of GFP^+^ cells sorted by fluorescence-activated cell sorting (FACS) increased massively (5- to 8-fold) after allografting (Fig. 8D-F). As in *brat* tumors *in situ*, all the GFP^+^ clones analyzed by FISH in allografted tumors (*n*=70) retained the Y-*GAL80* chromosome (Fig. 8C). These results show that Illuminati is enhanced in *brat* tumors. To investigate the fate of GFP^+^ and GFP^−^ cells in the tumor, we allografted samples that contained only GFP^+^ or GFP^−^ cells purified by FACS from a *Tub-GAL80^Y-29.7^ brat^mut^* allograft at T0 (Fig. 8F), and used FACS again to quantify the fraction of GFP^−^ and GFP^+^ cells in the resulting T1 allograft (Fig. 8D). We found that most (90.0%) of the cells in these T1 tumors that developed from FACS-sorted GFP^+^ T0 cells remained GFP^+^, strongly suggesting that the effect that stabilizes GFP expression in Illuminati NBs remains under malignant growth conditions at time points that are well beyond the constraints of normal larval development (Fig. 8F,G). In addition, we found that a significant fraction (20.9%) of the cells in T1 tumors that develop upon allograft of FACS-sorted GFP^−^ T0 cells became GFP^+^ (Fig. 8F,H), thus showing that *de novo* Illuminati events occur in T1 allografts. This time period corresponds roughly to about 3 weeks after the tumor started to develop in the larva.

Taken together, our results reveal that Illuminati is strongly enhanced during *brat* tumor growth, which in turn suggests that this new phenomenon may represent a previously unsuspected type of chromatin instability maintained in neural SC-derived tumor cells.

## DISCUSSION

We have generated a collection of *Drosophila* lines carrying a series of *GAL80* transgenes inserted in all major chromosomes, which can be used as sensors of GI. All these lines behave as expected, efficiently repressing *GAL4::UAS-GFP* in the testes, ovaries and imaginal discs. In larval brains, however, we found an unexpected number of fluorescent clones that, in most cases, originate from central brain NBs. Most of these clones retain the original wild-type *GAL80* transgene, hence ruling out GI and strongly suggesting an epigenetic origin. We have named this new type of gene expression plasticity ‘Illuminati’.

We do not know the molecular mechanism of Illuminati, but we know that it partially overlaps with PEV and Gal4::UAS variegation; although some suppressors of PEV and Gal4::UAS variegation have a mild suppressor effect on Illuminati, others have none, and some enhancers of PEV and Gal4::UAS variegation are Illuminati suppressors. The effect of temperature further substantiates the considerable difference between Illuminati and PEV. These results also reveal that, like PEV and Gal4::UAS variegation, Illuminati is controlled by key chromatin remodeling proteins. Particularly relevant among them is Su(var)2-10, which we have found to be a strong dominant suppressor of Illuminati. Su(Var)2-10 has been recently shown to be a SUMO ligase that controls H3K9 trimethylation, influencing the expression of heterochromatin and euchromatin regions (Ninova et al., 2020a,b). Through this activity, Su(var)2-10 could play a key role in stabilizing the epigenetic shift that represses Gal80 and in maintaining this inhibitory status through successive cell cycles. Also related to this activity, it will be interesting to investigate whether GFP fluorescence intensity correlates with H3K9 patterns in central brain NBs. Indeed, like for PEV and Gal4::UAS variegation, straightforward screening for dominant modifier genes that affect Illuminati frequency and/or cell-type specificity will pave the way to uncovering the underlying molecular mechanisms.

The special sensitivity of central brain NBs is intriguing. It could reflect the fact that SCs and early progenitor cells are less able than more differentiated cells to accurately transmit non-genetic information to their progeny (Lee et al., 2017; Skora and Spradling, 2010), a feature that has been proposed to be required for NB competence to generate different types of neurons (Cleary and Doe, 2006; Pearson and Doe, 2003). However, the extremely low rate of Illuminati in medulla and ventral nerve cord NBs, as well as in the somatic and germline SCs of testes and ovaries, shows that Illuminati is not a general feature of SCs. Besides its relevance in terms of epigenetic inheritance and neural stem cell development, there are two important practical considerations to be derived from the discovery of Illuminati. The first is that as far as larval brains, and particularly central brain NBs are concerned, a fraction of the clones generated by techniques based on the loss of *GAL80*, like MARCM and ‘gypsy-trap’ (Lee and Luo, 1999; Li et al., 2013), are bound to be Illuminati clones in which recombination (MARCM) or gypsy integration (gypsy-trap) have not taken place. The second practical consideration relates to the potential of Illuminati as a method to generate random clones in central brain NBs expressing any UAS-driven sequence of interest. Taking advantage of the collection of *GAL80* insertions reported here, it is possible to design experiments such that the expected average number of clones can be predetermined, from many to just one clone per brain. Moreover, unlike recombination-based methods that only allow for binary on/off conditions, Illuminati clones present a rather wide dynamic range of expression levels, which may reveal dosage-dependent effects that would pass unnoticed in conventional clones.

In summary, the discovery of Illuminati identifies a previously unappreciated form of gene expression plasticity that operates during normal development in *Drosophila* neural stem cells and is strongly enhanced in at least one type of neural stem cell-derived malignant neoplastic tumor. Like other types of gene expression variegation described in *Drosophila*, further investigation into the molecular basis of Illuminati may provide a valuable means towards understanding how alterations in the chromatin-based machinery of epigenetic inheritance contribute to neural stem cell development under normal and disease conditions.

## MATERIALS AND METHODS

### Fly husbandry and fly stocks

For most experiments, flies were raised in plastic vials containing homemade standard *Drosophila* rich culture medium [0.75% agar, 3.5% organic wheat flour, 5% yeast, 5.5% sugar, 2.5% nipagin, 1% penicillin-streptomycin (Gibco, 15140), and 0.4% propanic acid]. Fly stocks were maintained at 22°C and experimental crosses at 22°C or 25°C. For the food restriction experiment, flies were raised on homemade protein-poor medium (0.75% agar, 7% cornmeal, 1.4% yeast, 5.2% sugar and 1.4% nipagin) at 22°C and compared to flies raised on homemade standard rich medium at 22°C. For temperature variation experiments, flies were laying eggs for 24 h and the tubes containing progeny were maintained at 18°C, 22°C, 25°C or 29°C for 7, 5, 5 or 4 days prior dissection, respectively. For MARCM experiments to estimate proliferation in wing discs and brain lobes, females of the genotype *hsFLP Tub-GAL80^X-1C2^ neoFRT19A; Tub-GAL4 UAS-cD8-GFP/CyO S Tb* were crossed to *neoFRT19A* males; crosses were kept at 25°C, heat-shocked for 1.5 h at 37°C, 48-72 h after egg laying, and brains and wing discs of female larvae were dissected at 96-120 h after egg laying. For the Sas4 MARCM experiments, fly crosses were kept at 22°C. L2 progenies were heat-shocked 1 h at 37°C in a water bath and maintained at 22°C for 48±12 h before dissection. The following stocks were used: *Su(var)3-3^DeltaN^/TM6B Tb^1^ Hu^1^* from the laboratory of Ming-Chia Lee (Department of Life Sciences and Institute of Genome Sciences, Yang-Ming University, Taiwan), +*; P{EPgy2}Six4^EY09833^/TSTLR Cy Tb^1^* (BDSC, #16956); *Parp^CH1^ ry^506^/TM6 Tb^1^* (BDSC, #81887), *y^1^ w^67c23^ P{y^+mDint2^ w^+mC^=EPgy2}CoRest^EY14216^* (BDSC, #20793), *+; mus312^D1^/TSTLR Cy Tb^1^* (BDSC, #329), w*; Su(var)2-10^1^/CyO-TbA (BDSC, #6236), *In(1)w^m4h^; Su(var)2-10^2^/CyO* (BDSC, #6235), *PBac{Su(var)2-10-GFP}* (BDSC, #64795) and w*;Su(var)3-9^1^/TM6B Tb^1^ Hu^1^ (BDSC, #6209) and *E(var)8^48g^/CyO-TbA* (BDSC, #6231).

### Establishment of *Tub-GAL80 Drosophila* lines

For the φC31-recombination attB P[acman] system for *Tub-GAL80* insertions on chromosome X, II and III, the plasmid containing a codon-optimized *GAL80* sequence driven by a tubulin promoter corresponds to the combination of *pattB-tubP-SV40* – generated by Lee and Luo (1999) – with the codon-optimized *GAL80* sequence from *pBPGAL80Uw-6* (Addgene #26236, deposited by Gerald Rubin) (Pfeiffer et al., 2010). Then, the plasmid was inserted in a P[acman] vector and sent to the Bestgene company to integrate it into the *Drosophila* genome at specific insertion sites using the φC31 integrase-mediated transgenesis system.

To obtain *Drosophila* recombinants carrying two copies of *Tub-GAL80* on the same chromosome, we used female meiotic recombination and selected recombination events based on the fly eye color, a method widely used to generate *Drosophila* recombinants. Indeed, all *Tub-GAL80* lines were associated with the white^+^ transgene-expressing marker, which is used as a marker for efficient transgene insertion as it confers a yellow-to-red eye color. Simply, in the presence of two copies for efficient recombination, fly eyes display a strong red color.

The *P{w^+^, tubP-GAL80}LL1* transgene terminally located on the X chromosome (Lee and Luo, 1999) was recombined onto a *y w f-*marked *C(1;Y)* chromosome. The resulting *C(1;Y) y+ P{w+, tubP-GAL80}LL1 w f* chromosome was mutagenized with 4000 R of X-rays to induce deletions that remove most of the X chromosome.

### Deep sequencing

Illumina reads were aligned by BWA software (version 0.7.10; Li and Durbin, 2009) using default options and data were analyzed using SAM tools (version 0.1.19; Li et al., 2009). Whole-genome alignment was performed using the *Drosophila melanogaster* release 6.02 as reference (http://fb2017_05.flybase.org/static_pages/archive/releasenotes/FB2014_05/release_notes_fb2014_05.html).

### Immunofluorescence of *Drosophila* larval whole-mount tissues

Wandering third-instar larval (L3) brains and imaginal discs were dissected in fresh 10× PBS (VWR, L182-10) and fixed for 30 min at room temperature (RT) in 4% paraformaldehyde (PFA) (Electron Microscopy Sciences, 15710) diluted in PBS. Fixed tissues were washed and permeabilized three times for 15 min in PBS with 0.3% Triton X-100 (Euromedex, 2000-C) (PBST3) or 0.1% Triton X-100 (PBST1). For antibody staining, larval tissues were incubated in primary antibodies diluted in PBST3 or PBST1 overnight at 4°C in a humid chamber. After three 15 min washes in PBST3 or PBST1, tissues were incubated in secondary antibodies diluted in PBST3 or PBST1 overnight at 4°C and protected from light in a humid chamber. Tissues were then washed three times for 15 min in PBST3 or PBST1, rinsed in PBS and mounted between slides (Thermo Fisher Scientific, AA00008232E00MNT10) and 12-mm circular cover glasses (Marienfield Superior, 0111520) with 5 µl of homemade mounting medium (1.25% n-propyl gallate, 75% glycerol and 25% H_2_O).

For GFP labeling of larval brains, imaginal discs and ovaries, as well as of adult testes and ovaries, two different protocols were used. In the first protocol, overnight incubation at 4°C with GFP booster Alexa Fluor 488 (1:250, Chromotek, gb2AF488), Alexa Fluor 647 phalloidin (1:250, Thermo Fisher Scientific, A-22287) and DAPI (1:1000, Thermo Fisher Scientific, 62248) was performed, followed by three 15 min washes in PBST3, a rinse in PBS and mounting. If antibodies were used, samples were incubated with the primary antibodies 4°C overnight (see list below) and with the secondary antibodies for 4 h at 25°C. For GFP labeling of larval ovaries, we used a similar protocol as that for brain fixation described above. Briefly, ovaries were dissected in PBS and fixed in 4% PFA, followed by three washes in PBST1. Incubation with GFP booster was performed in PBST1 with 0.3% normal goat serum overnight. After three washes and incubation with DAPI, ovaries were mounted in 4.5-8 μl drops of mounting medium.

In the second protocol, brains and imaginal discs of wandering L3 larvae and testes of 0- to 4-day-old adult males as well as ovaries of 2- to 10-day-old adult females were dissected in fresh PBS and fixed at RT in 4% PFA diluted in PBST1. The fixation times depended on the tissues, with 15 min for testes, 20 min for larval discs and brains, and 30 min for ovaries. The fixed tissues were washed three times for 10 min in PBS, permeabilized for 1 h in PBST3, washed for 10 min in PBS, stained with DAPI (1:1000) for 20 min, washed again for 10 min in PBS and mounted in Vectashield (Vector Laboratories, H-1000-10).

The following primary antibodies were used in this study: chicken anti-GFP (1:1000, ab13970, Abcam), guinea pig anti-Deadpan (Dpn) (1:1000, gifted by J. Skeath, Washington University School of Medicine, St Louis, MO, USA; Gogendeau et al., 2015), mouse anti-Prospero [1:500, MR1A, Developmental Studies Hybridoma Bank (DSHB)], rat anti-Elav (1:100, 7EA10, DSHB), mouse anti-Repo (1:500, 8D15, DSHB), rabbit anti-Sas4 (1:500, Basto et al., 2006), guinea pig anti-Centrosomin (Cnn) (1:1000, Lucas and Raff, 2007). The following secondary antibodies (1:250) were used in this study: goat anti-chicken IgY Alexa Fluor 488 (Thermo Fisher Scientific, A-11039), goat anti-rat IgG Alexa Fluor 546 (Thermo Fisher Scientific, A-11081), donkey anti-rabbit IgG Alexa Fluor 568 (Thermo Fisher Scientific, A-10042), goat anti-mouse IgG Alexa Fluor 546 (Thermo Fisher Scientific, A-11030) and goat anti-guinea pig IgG Alexa Fluor 647 (Thermo Fisher Scientific, A-21450).

Images were acquired with 40× (NA 1.25), 63× (NA 1.32) or 100× (NA 1.4) oil objectives on a wide-field inverted spinning-disk confocal Gattaca/Nikon microscope (a Yokogawa CSU-W1 spinning head mounted on a Nikon Ti-E inverted microscope, equipped with a camera complementary metal-oxide semiconductor 1200×1200 Prime95B; Photometrics). Intervals for *z*-stack acquisitions were set to 0.5-1.5 µm using Metamorph software.

### Live imaging of *Drosophila* larval brains

Mid second-instar larval (L2) brains were dissected in Schneider's *Drosophila* medium (Gibco, 21720024) supplemented with 10% heat-inactivated fetal bovine serum (Gibco, 10500), penicillin (100 U/ml) and streptomycin (100 µg/ml). Several brains were placed in 10 µl of medium on a glass-bottomed dish (Dutcher, 627870), covered with a permeable membrane (YSI 066155), and sealed around the membrane borders with oil 10 S Voltalef (VWR Chemicals). Images were acquired with 60× oil objectives (NA 1.4) on two microscopes: an inverted spinning-disk confocal Roper/Nikon microscope (a Yokogawa CSU-X1 spinning head mounted on a Nikon Ti-E inverted microscope, equipped with a camera EMCCD 512×512 Evolve; Photometrics) and the wide-field inverted spinning-disk confocal Gattaca/Nikon microscope (a Yokogawa CSU-W1 spinning head mounted on a Nikon Ti-E inverted microscope, equipped with a camera complementary metal-oxide semiconductor 1200×1200 Prime95B; Photometrics), controlled by Metamorph software. For both microscopes, images were acquired at time intervals spanning 30 min and 50 z-stacks of 1.5 µm.

### DNA FISH

After fixation, permeabilization and overnight incubation with GFP booster, brains were washed three times for 15 min in PBST3 and fixed a second time for 30 min in 4% PFA. Then, brains were rinsed three times in PBS, washed once for 5 min in 2× saline sodium citrate (SSCT) (Euromedex, EU0300-A) with 0.1% Tween-20 (Sigma-Aldrich, P1379) diluted in water, once for 5 min in 2× SSCT/50% formamide (Sigma-Aldrich, 47671), transferred to pre-warmed 2× SSCT/50% formamide and pre-hybridized for 3 min at 92°C. DNA probes diluted in the hybridization buffer [20% dextran sulfate (Sigma-Aldrich, D8906), 2× SSCT, 50% formamide, 0.5 mg/ml salmon DNA sperm (Sigma-Aldrich, D1626)] were denatured at 92°C. After removal of the supernatant, brains were incubated in the DNA probe solution and hybridized for 5 min at 92°C and overnight at 37°C. Brains were then rinsed at RT, washed for 10 min at 60°C and again for 5 min at RT in 2× SSCT. Finally, after a rinse in PBS, brains were mounted as described below. DNA probes (Sigma-Aldrich) used in this study were against chromosomes X (80 ng/µl), II (40 ng/µl) and III (80 ng/µl). FISH for the Y chromosome was performed with a probe to detect the AATAC repeat, one of the Y-specific satellites of the *Drosophila* genome (Bonaccorsi and Lohe, 1991). Chromosome spreads of mitotic chromosomes (karyotypes) were prepared and stained with DAPI following standard procedures (Fanti and Pimpinelli, 2004). Hybridization conditions were as described in Dernburg (2011).

### RNA FISH

L3 brains were dissected in fresh PBS, fixed for 30 min in 4% formaldehyde (Electron Microscopy Sciences, 15686) and washed and permeabilized in PBS with 0.3% Tween-20 (PBSTw). Brains were incubated with GFP booster and phalloidin diluted in PBSTw at 4°C overnight in a humid chamber. After three 15 min washes in PBSTw, RNA hybridization was performed as described by Yang et al. (2017). RNA probes against *GAL80* were designed by the Biosearch Technologies technical support team (https://www.biosearchtech.com/) and labeled with Quasar 570.

### Allografts

Allografts were performed as described in Rossi and Gonzalez (2015).

### Generation of *brat* tumors in Illuminati clones

Males of the genotype y^1^ sc* v^1^ sev^1^; UAS-brat-RNAi (P{TRiP.HMS01121}attP2) (Ni et al., 2011) were crossed to females *Tub-GAL80^X-1C2^; Tub-GAL4 UAS-cD8::GFP/CyO, S, Tb* and incubated at 29°C. Five-day-old female larvae of the genotype *Tub-GAL80^X-1C2^/+; Tub-GAL4 UAS-cD8::GFP/+; UAS-brat-RNAi/+* were dissected and brain lobes were allografted into RFP-α-Tub female hosts. Tumors were cultured for 12-13 days at 29°C, flies were dissected and GFP^+^ tumor cells were prepared for DNA extraction.

### DNA extraction, PCR and sequencing

Genomic DNA was extracted from tumors using standard phenol/chloroform protocols and precipitated with isopropanol in the presence of Pellet Paint (Merck). The amplification was done using 2 ng of DNA and KOD hot start DNA polymerase (Merck). PCR fragments were sequenced by Eurofins Genomics. Primers used for PCR and sequencing are listed in Tables S1 and S2.

### FACS

FACS was performed as in Castellanos et al. (2008). Briefly, brain lobes or allograft-derived tumors were dissected in Rinaldini's solution (800 mg NaCl, 20 mg KCl, 5 mg NaH_2_PO_4_, 100 mg NaHCO_3_, 100 mg glucose in 100 ml distilled H_2_O) and incubated at 30°C for 60 min in dissociation solution made of complemented Schneider's medium (5 ml fetal bovine serum, 0.1 ml insulin, 1 ml PenStrep, 5 ml L-glutamine and 0.4 ml L-glutathione in 37.85 ml Schneider's medium) supplemented with 1 mg/ml papain and 1 mg/ml collagenase. The dissociation solution was then replaced with complemented Schneider's medium and tissues were disrupted by repeated pipetting. Flow cytometry was carried out using a FacsAria I SORP sorter (Beckton Dickinson) at the Cytometry Unit, Centres Científics i Tecnològics (CCiT) of the University of Barcelona. 488 nm and 561 nm excitation laser beams were used for forward and side scatter (FSC and SSC), respectively. Cells were gated according to their FSC versus SSC parameters; doublets were excluded using TOF parameter. Brain lobes from *w1118* larvae and *Tub-GAL4 UAS-CD8::GFP* heterozygous larvae were used to gate GFP-negative and -positive populations, respectively. A 70 μm nozzle was used to sort GFP^+^ or GFP^−^ cells. FlowJo software was used to visualize the flow cytometry data (FlowJo 10.7.2, https://www.flowjo.com/citing-flowjo).

## Supplementary Material

10.1242/develop.200808_sup1 Supplementary information
