# Supplementary material for: Illuminati: a form of gene expression plasticity in Drosophila neural stem cells
Source: Development. 2022 Nov 18;149(22):dev200808. doi: 10.1242/dev.200808 (PMC9845751; doi:10.1242/dev.200808)
Supplement: Supplementary information [file develop-149-200808-s1.pdf]

Development: doi:10.1242/dev.200808: Supplementary information

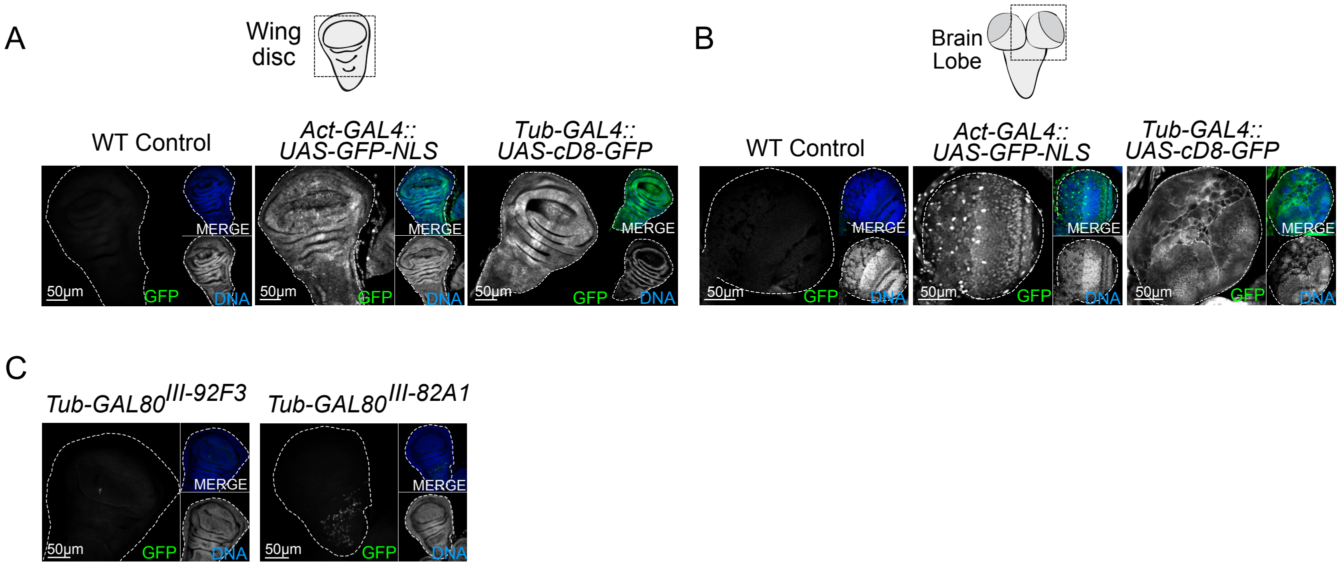

D

Average ±SD GFP+ cells or clusters in 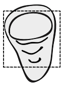

24.1	14.1	29.7	22.1	1C2	5B8	19E7	22A	25A3	28E7	43A1	47C6	53B2	59D3	62E1	65B2	70C3	75A10	76A2	82A1	85A2	89E	92F3	96F3	99F
0	0	0.02 ±0.1	0	0.05 ±0.2	0	0	0	0.06 ±0.3	0.16 ±0.4	0	0	0.18 ±0.4	0	0.09 ±0.3	0.06 ±0.3	0	0	0	0.25 ±0.5	0	0	0.13 ±0.3	0	0
**14**	**10**	**60**	**12**	**39**	**17**	**13**	**20**	**16**	**12**	**15**	**18**	**11**	**11**	**12**	**15**	**11**	**9**	**18**	**12**	**7**	**16**	**16**	**13**	**10**

E

Average ±SD GFP+ cells or clusters in 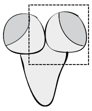

24.1	14.1	29.7	22.1	1C2	5B8	19E7	22A	25A3	28E7	43A1	47C6	53B2	59D3	62E1	65B2	70C3	75A10	76A2	82A1	85A2	89E	92F3	96F3	99F
1.0 ±0.9	1.3 ±1.3	0.7 ±0.9	1.6 ±1.0	0.5 ±0.2	50.3 ±7.6	2.6 ±1.9	0.5± 1.0	2.6 ±4.6	5.7 ±6.6	11.9 ±9.8	4.6 ±4.5	2.5 ±3.2	1.8 ±1.7	5.2 ±7.6	1.8 ±3.0	9.9 ±6.5	1.4 ±1.6	28.0 ±18.2	9.1 ±12.9	2.6 ±3.5	0.6 ±1.4	6.3 ±6.9	3.0 ±2.2	1.3 ±2.2
**36**	**14**	**32**	**19**	**43**	**43**	**32**	**28**	**27**	**17**	**19**	**27**	**22**	**18**	**19**	**22**	**25**	**26**	**26**	**20**	**13**	**27**	**24**	**25**	**23**

Fig. S1. Analysis of Control and *Tub-GAL80* wing discs and brains.

(A-C) Images of whole mount tissues labelled for GFP (grey and green in large and small insets respectively) and DNA (blue and grey in small insets) of the indicated genotypes. White dotted lines surround tissues. (D-E) Table summarizing the average ±SD of green cells in each line in wing discs (D) and brain lobes. Figures in bold represent the number of samples analyzed.

Development • Supplementary information

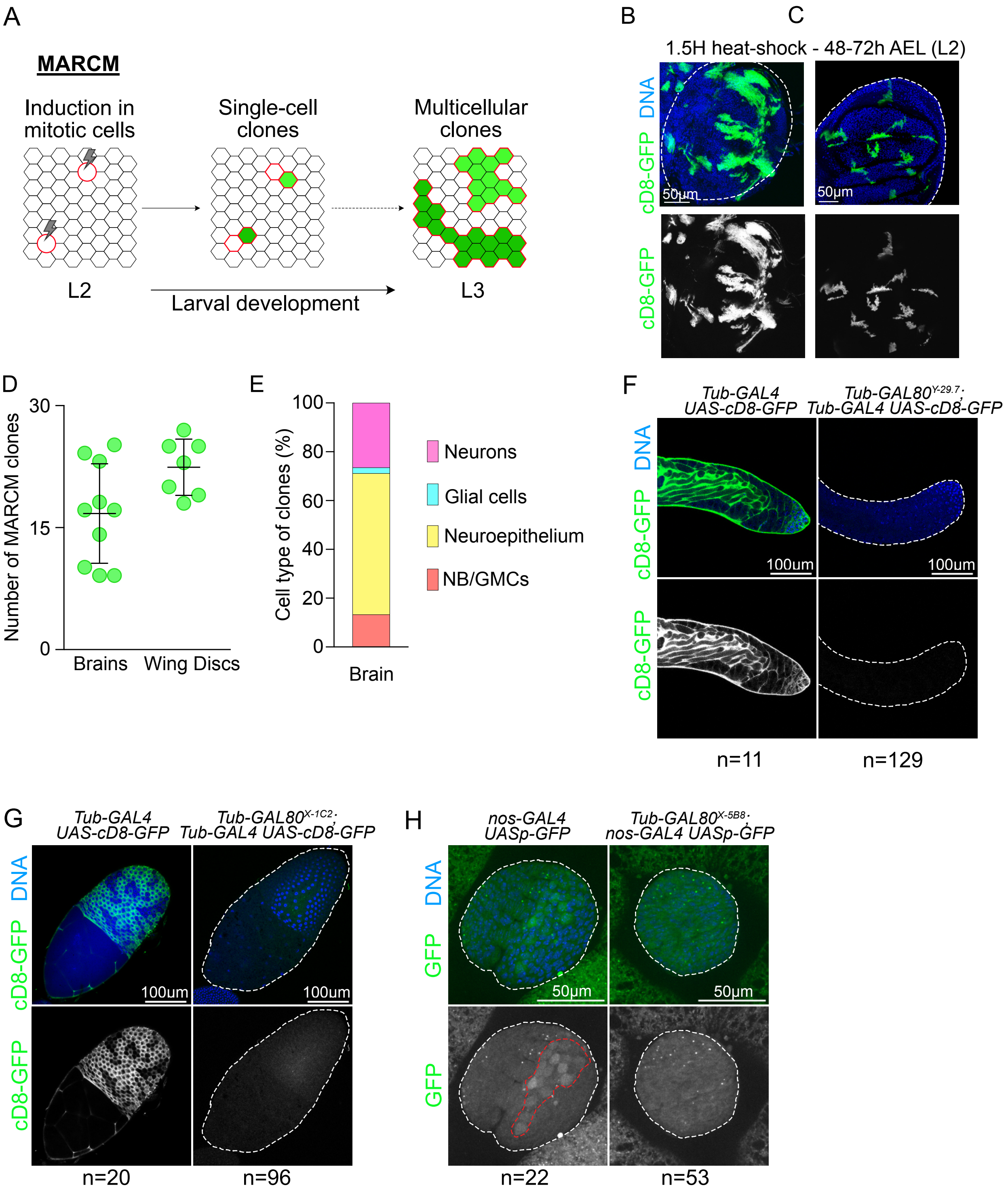

Development • Supplementary information

Fig. S2. The presence of high level of GFP+ cells in the brain is not explained by a higher proliferation rate.

(A) Schematic representation of the clone induction with MARCM system during development as a proxy to determine the frequency of mitosis and proliferation in tissues. (B-C) Images of MARCM clones in a brain lobe (B) and a wing disc labeled for GFP (grey and green) and DNA (blue). White dotted lines surround tissues. (D) Dot plot showing the number of MARCM clones in brain lobes (n=10 BLs) and wing discs (n=7 Wing discs). Error bars correspond to the means ± SD. (E) Graph bar showing the percentage of cells showing GFP+ signals for each cell type of the brain lobe: NB/GMCs clusters (red), cells from the optic lobe (yellow), individual glial cells (cyan blue) and individual neurons (pink). (F-H) Images of adult male testis (F), adult egg chambers (G) and larval ovaries (H) from individuals of the indicated genotypes. White dotted lines surround tissues and the red dashed line in (H) surrounds the GFP+ GSCs.

Development • Supplementary information

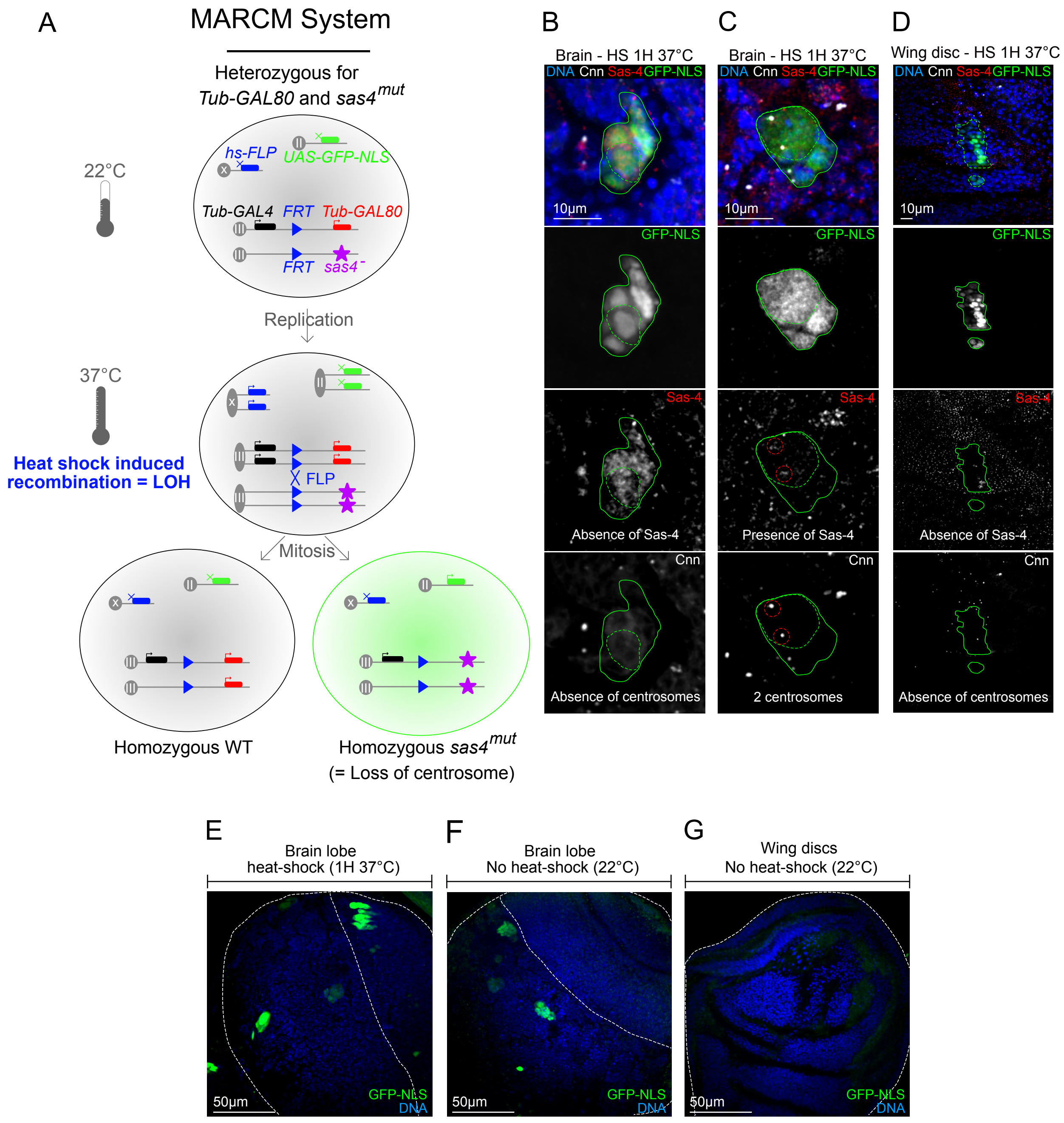

Development • Supplementary information

Fig. S3. Analysis of *Tub-GAL80* lines alerts its use for MARCM analysis.

(A) Schematic representation of the MARCM system to induce labeled mutant clones. After recombination of FRT sites by the heat-induced FLP recombinase, the daughter cells lose heterozygosity. One cell becomes homozygous *sas4*^{mut} and is labeled with GFP due to the loss of the *Tub-GAL80* sequence. The other cell becomes homozygous WT and it is unlabeled. (B-D) Images of GFP+ clones in (B-C) brain lobes and (D) wing disc of *hs-FLP/+, UAS-GFP-NLS/+; Tub-GAL4,FRT82B,Tub-GAL80/FRT82B,sas4*^{mut} flies heat-shocked at 37°C for 1 hour and labeled with antibodies against GFP (green and grey), *Sas-4* (red and grey), Cnn (grey) and with DAPI for DNA (blue). Green continuous and dotted lines surround GFP+ clones and NBs, respectively. (B) Sas4 mutant GFP+ NB without centrosomes. (C) Wild type GFP+ NB with two centrosomes. (D) Sas4 mutant GFP+ clones in the WD. (E-G) Images of whole mount (E-F) brain lobes and (G) wing discs labeled for GFP (green) and with DAPI for DNA (blue). (E) Presence of GFP+ clones in brain lobes after 1 hour of heat-shocked at 37°C. (F-G) In the absence of heat-shock, (F) brain lobes present GFP+ clones, in contrast to (G) wing discs that are GFP_.

Development • Supplementary information

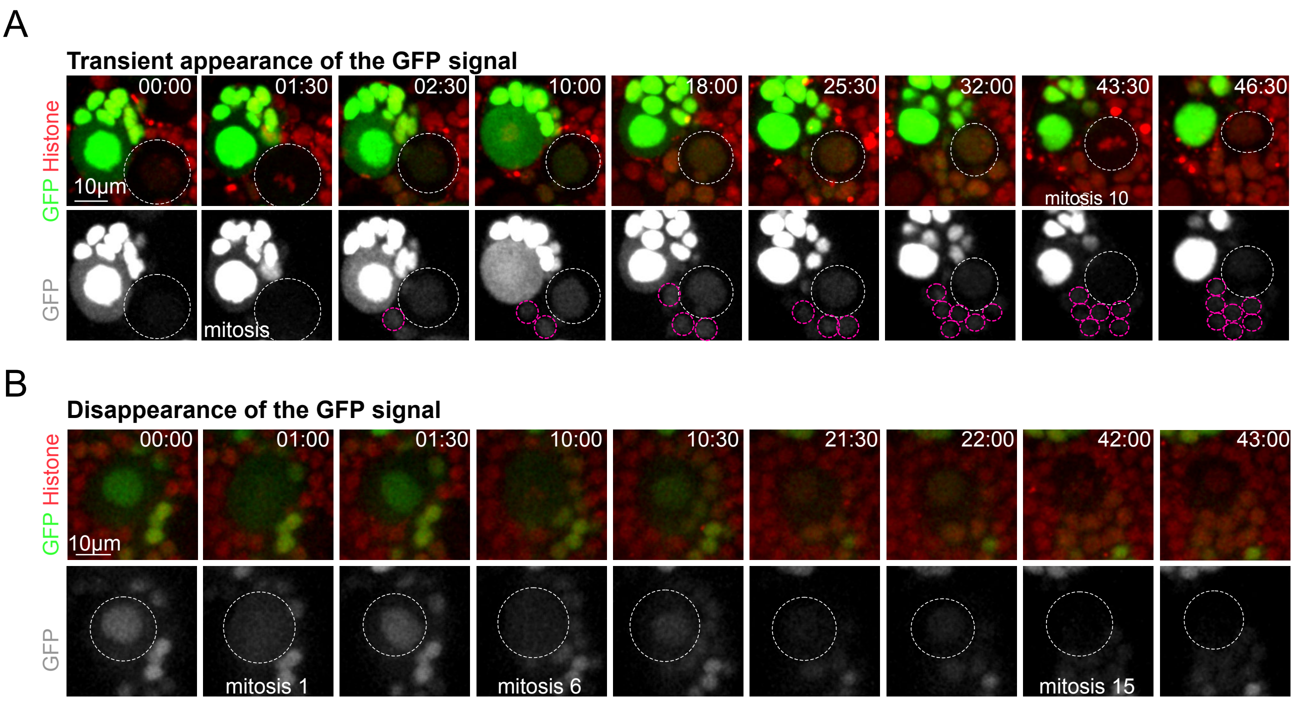

Fig. S4. Illuminati expression is dynamic and reversible in extremely rare cases. (A-B) Stills of time-lapse movies of mitotic NBs expressing *2xTub-GAL80*$^{X\text{-}5B8,19E7}$, *GAL4::GFP-NLS* (green) and *histone-RFP* (red) to monitor GFP and chromosome dynamics. White and pink dotted circles surround NBs and GMCs, respectively. GFP signal is dynamic in rare cases: (A) the transient appearance or (B) the disappearance of the GFP signal.

Development • Supplementary information

Table S1. PCR primers

Primer Name	Sense	Sequence
GAL4.F	Forward	CCAGTGACAATACCAAGAAGCACACC
GAL4.R	Reverse	CACGATGCACAGTTGAAGTGAACTTG
PCR1.F	Forward	TCCAGACATGATAAGATACATTGATGAG
PCR1.R	Reverse	CCGCTAGCCAATGGACAACA
PCR2.F	Forward	CCACTGCTCCCATTCATCAG
PCR2.R	Reverse	GGACTACAACAAGAGATCTTCGGTC
PCR3.F	Forward	AGTATGGCGGGGTAATGTGTCTTG
PCR3.R	Reverse	CCGCAAAAATGGGTTTTATTAACTTACATAC

Table S2. Sequencing primers

Primer Name	Sense	Sequence
PCR1.1000F	Forward	TGTTGTCCATTGGCTAGCGG
PCR1.1500F	Forward	TTAATGTCGCCGATATAGCC
PCR1.1400R	Reverse	ATCGAGATTGCTGGAAATGG
PCR1.500R	Reverse	CTGATGAATGGGAGCAGTGG
PCR2.500F	Forward	CCGCCATTTTGAGAAAAAGC
PCR2.1000F	Forward	GGTTGCTAAAGTGGGCCAAC
PCR2.1500F	Forward	CCGTCCGCGAAAGACCAGTG
PCR2.2000F	Forward	AGACTAAAGCCCGCTGATCG
PCR2.2500F	Forward	ACTTACGCAGAAGTGCAGTC
PCR2.300R	Reverse	GAGAGAGTAAAATTCCGGCG
PCR2.800R	Reverse	CAGCCCGCTTTCCACATTTC
PCR2.1300R	Reverse	GTGACCATGAGTAGGAGTTC
PCR2.1800R	Reverse	GCCTTTGTTCGACTGCCAAT
PCR2.2300R	Reverse	CTGGCTGATTGTTGGGATTG

Development • Supplementary information
