# Supplementary material for: Illuminati: a form of gene expression plasticity in Drosophila neural stem cells
Source: Development. 2022 Nov 18;149(22):dev200808. doi: 10.1242/dev.200808 (PMC9845751; doi:10.1242/dev.200808)
Supplement: Supplementary file 1 [file develop-149-200808_review_history.pdf]

Illuminati: a form of gene expression plasticity in *Drosophila* neural stem cells

Alix Goupil, Jan Peter Heinen, Riham Salame, Fabrizio Rossi, Jose Reina, Carole Pennetier, Anthony Simon, Patricia Skorski, Anxela Louzao, Allison J. Bardin, Renata Basto and Cayetano Gonzalez
DOI: 10.1242/dev.200808

Editor: Thomas Lecuit

Review timeline

Original submission:	29 March 2022
Editorial decision:	21 June 2022
First revision received:	15 September 2022
Accepted:	6 October 2022

Original submission

First decision letter

MS ID#: DEVELOP/2022/200808

MS TITLE: Illuminati, a novel form of gene expression plasticity in Drosophila neural stem cells

AUTHORS: Alix Goupil, Jan Peter Heinen, Riham Salame, Fabrizio Rossi, Jose Reina, Carole Pennetier, Anthony Simon, Anxela Louzao, Allison Bardin, Renata Basto, and Cayetano Gonzalez

I sine rely apologize for the delay. I have now received all the referees reports on the above manuscript, and have reached a decision. The referees' comments are appended below, or you can access them online: please go to BenchPress and click on the 'Manuscripts with Decisions' queue in the Author Area.

The overall evaluation is contrasted. While both referees find the work very interesting, one of them is concerned by the lack of mechanistic understanding. I looked at the manuscript carefully and I think we could publish your manuscript after you address point 1 from Reviewer 1 (test existence of Illminati in other stem cells) and comments from Reviewer 2. If it would be helpful, you are welcome to contact us to discuss your revision in greater detail. Please send us a point-by-point response indicating your plans for addressing the referee’s comments, and we will look over this and provide further guidance.

Reviewer 1

Advance summary and potential significance to field

Genomic instability can result in profound effects on gene expression and consequently underpins many types of cancer. The authors set out to develop a genetic context that would allow them to probe for the frequency of genomic instability (GI) in developing tissues. This is based on the loss of the repressor Gal80 and results in cells acquiring GFP expression. Using the clever strains they generated, they observed that one tissue, the wing disc, exhibited very little GFP, indicating that GI is rare. In contrast brains exhibited much higher frequencies of fluorescence. Probing more deeply, their results show that the GFP expression is not due to GI instability, as the Gal80 gene is

© 2022. Published by The Company of Biologists under the terms of the Creative Commons Attribution License (https://creativecommons.org/licenses/by/4.0/).

still present. Instead it appears to be a novel epigenetic phenomenom. They coin the term "illuminati" to describe this and show that it is sensitive to some environmental challenges.

This is a very thorough study of a genetic phenomenom that results in spontaneous inactivation of Gal80 in neuroblast lineages. The experiments are all well-conducted and analyzed. However, it is unclear what mechanism underpins the Gal80 inactivation and what this tell us about developmental gene regulation. The results will be of interest to Drosophila researchers as they reveal some inherrant problems with currently used techniques. In summary, the results are very novel but how they contribute to our understanding of developmental mechanisms is less clear and because of that the work is currently unlikely to be of a broad interest to the developmental biology community.

Comments for the author

The observations are interesting and the experiments have been very well conducted. The limitation is that they report a phenomenom without insight into the mechanisms or an indication of how widespread this might be. To be of interest for a wider readership would require extensions of the study, rather than revisions.
For example:
In the discussion they propose that "illuminati" may be a feature of stem cells, but at present they contrast only the wing disc epithelium and the larval brain. Are similar effects seen in other stem cells?
Similarly, they also refer to some previous work in follicle cells that revealed a chromatin based mechanism that leads to variegation in Gal4 expression. Is there a similar variagation in Gal80 loss in follicle cells?-- this may provide an opportunity to probe the mechanisms, based on the previous work from the Spradling lab.
The assay uses an artificial system that is powerful for probing the GI but may not reflect what happens with normal endogenous genes. Is there evidence that any developmental repressors become inactivated in stem cells in a similar way, could they devises a system that relies on an endogenous repressor to test this?
Their mini-screen of some chromatin regulators suggests that Su(var)10-2 is involved in the shutting down of Gal80. To tie this in more directly ChIP or ATAC approaches could be used to probe whether the Gal80 is in heterochromatin, given that they can use genetic tricks to obtain reasonable yields of the "illuminati" cells. And/or they could test some of the other genes implicated in working with Su(var)10-2 such as SetDB1/Wde.
Minor comment: The current manuscript is not very accessibile for a more general reader, not expert in fly genetics. For example, the abstract is not geared towards a more diverse readership. More information about the homologue of Su(var)10-2 (and the other regulators tested) would also be helpful.

<u>Reviewer 2</u>

Advance summary and potential significance to field

The authors identifies a novel form of functional instability during development that leads to the stochastic inactivation of a reporter gene that is then transmitted mitotically. The discovery described here rests on the use of a sophisticated fluorescence-based method developed in the Drosophila system to identify the birth and the behavior of a mutant cell clones within a developing tissue. Because the inactivation of the reporter gene lead to the activation of GFP in the cell and its descendants, the authors name this phenomenon “Illuminati”. The initial aim of this study was to investigate the phenomenon of genetic instability. Surprisingly however, the authors do not find any evidence of genetic instability in the clones of cells in which illuminati occurred. Given the observation that illuminati is sensitive to mutations affecting position-effect- variegation, the authors propose that it reflects some kind of chromatin based epigenetic inactivation. Illuminati occurs in the central brain neural stem cells and their resulting lineages. It should be finally emphasized that Illuminati does not seem to depend on the genomic environment in which the reporter genes is sitting as they observed it in a total of 20 Drosophila transgenic lines with the GAL80 reporter inserted at different genomic regions on all 4 chromosomes.

© 2022. Published by The Company of Biologists under the terms of the Creative Commons Attribution License (https://creativecommons.org/licenses/by/4.0/).

The observations described in this work are very intriguing and interesting and could only be made in the model organism Drosophila and its powerful toolbox for genetic manipulation (especially the bipartite Gal4/UAS-GFP combined with the repressor GAL80). It is probably a more general phenomenon as it has been previously observed that reporter genes stably integrated in tissue cultured cells are often silenced if a selective pressure is not exerted (see for example Pikaart et al. 1998; https://pubmed.ncbi.nlm.nih.gov/9744862/). In the living larva however illuminati only occurs in the neural stem cells. Whether this observation reflects plasticity in the genetic program of this tissue to generate multiple cell fates is highly speculative and is way beyond the scope of this article which to my opinion will be of a great interest to the wide field of genomic plasticity during development and disease.

Comments for the author

Some considerations for the future: I assume that all the components of the bipartite system are in heterozygous conditions in the analyzed larval brains. Otherwise my following comments do not make sense. The trigger leading to inactivation of the reporter gene could be direct, by aiming at the Tub-GAL80 transgene, or could be an indirect effect due to the inactivation of the neighboring chromatin. In the 1st case this would lead to heterozygocity, but in the 2nd case both homologs could be inactivated. I am wondering if the authors have thought of probing for the activity of the genes immediately adjacent to the integration platform that carry their Tub-GAL80. They could test by in situ hybridization to see if the neighboring gene(s) also become inactive in the Illuminati clones. Furthermore, it would be instructive to know the status of the white+ gene adjacent to the Tub-GAL80 transgene in the illuminati clones. . Finally, what happen in case when the Tub-GAL80 transgene is in homozygous conditions?

Minor comments

I am confused by the experiments reported in Supplementary Figure3, as I would expect that sas4 minus clones would fail to divide, unless there is perdurance of the SAS4 protein. Please explain Could have used the Bischof et al 102D line on the 4th.

First revision

<u>Author response to reviewers' comments</u>

Reviewer 1 Comments for the Author:

_In the discussion they propose that "illuminati" may be a feature of stem cells, but at present they contrast only the wing disc epithelium and the larval brain. Are similar effects seen in other stem cells?

To address this key question, we have analysed testis and ovaries that contain different types of somatic and germline SCs. The results from these experiments (new Supplementary Figure 2) reveal no evidence for illuminati in any of these SC types. These results show that illuminati is, to say the least, much less frequent in all the SCs of testis and ovaries than it is in the neural stem cells (neuroblasts) that populate the larval central brain. Therefore, illuminati does not appear to be a general feature of SCs, certainly not at the rate that it occurs in central brain neuroblasts.

_Similarly, they also refer to some previous work in follicle cells that revealed a chromatin based mechanism that leads to variegation in Gal4 expression. Is there a similar variagation in Gal80 loss in follicle cells?-- this may provide an opportunity to probe the mechanisms, based on the previous work from the Spradling lab.

As reported by the Spradling lab, the level of GFP expression variegation is conspicuous in Gal4/UAS-GFP follicle epithelia. That is of course in the absence of Gal80 (Supplementary Figure 2G). In the presence of Gal80 the Gal4/UAS-GFP system is fully repressed and neither follicle SCs

© 2022. Published by The Company of Biologists under the terms of the Creative Commons Attribution License (https://creativecommons.org/licenses/by/4.0/).

nor more differentiated cells express GFP. Therefore, Gal80 does not appear to variegate, at least not to any significant extent, in these cells.

Regarding possible functional links between the pathways that control Gal4/UAS-GFP variegation and Illuminati, our data show that they do share several regulatory elements, but some of them have opposite effects e.g. PARP and E(var)8 are suppressors of illuminati and enhancers of Gal4/UAS-GFP.

__The assay uses an artificial system that is powerful for probing the GI but may not reflect what happens with normal endogenous genes. Is there evidence that any developmental repressors become inactivated in stem cells in a similar way, could they devises a system that relies on an endogenous repressor to test this?

This is a very important issue that we have been struggling with since we first observed illuminati. Unfortunately, we still have not been able to devise a convincing experimental approach to tackle it. Having said that, as pointed out by Lee and Spradling (doi: 10.534/g3.116.036038) regarding Gal4/UAS variegation, even if illuminati reflected the artificial nature of the Gal80/Gal4/UAS system, the mechanisms that trigger illuminati and drive the inheritance of the Gal80 repressed state must rely on the normal Drosophila epigenetic machinery. Consequently, investigating the molecular bases of illuminati should reveal important components of this machinery.

__Their mini-screen of some chromatin regulators suggests that Su(var)10-2 is involved in the shutting down of Gal80. To tie this in more directly ChIP or ATAC approaches could be used to probe whether the Gal80 is in heterochromatin, given that they can use genetic tricks to obtain reasonable yields of the "illuminati" cells.

This is an excellent suggestion that we will start exploring in the near future.

_And/or they could test some of the other genes implicated in working with Su(var)2-10 such as SetDB1/Wde.

We will certainly investigate other factors involved with Su(var) 2-10 but rather than focusing exclusively on the Su(var)10 lead, we are currently engaged in a much larger screen that includes, among others, all chromatin modifier genes in Drosophila. From this study we hope to be able to obtain a comprehensive view of the gene networks that control illuminati.

_Minor comment: The current manuscript is not very accessibile for a more general reader, not expert in fly genetics. For example, the abstract is not geared towards a more diverse readership. More information about the homologue of Su(var)10-2 (and the other regulators tested) would also be helpful.

We have rephrased the text at different points throughout the manuscript to make it more easily accessible to a wider readership. As requested we have included some additional information about the homologues of Su(var)10-2 and the other regulators.

Reviewer 2 Comments for the Author:

__The observations described in this work are very intriguing and interesting and could only be made in the model organism Drosophila and its powerful toolbox for genetic manipulation (especially the bipartite Gal4/UAS-GFP combined with the repressor GAL80). It is probably a more general phenomenon as it has been previously observed that reporter genes stably integrated in tissue cultured cells are often silenced if a selective pressure is not exerted (see for example Pikaart et al. 1998; https://pubmed.ncbi.nlm.nih.gov/9744862/).

We thank the reviewer for pointing this out. It will be interesting to know if flanking Tub-Gal80 with insulators may affect illuminati.

© 2022. Published by The Company of Biologists under the terms of the Creative Commons Attribution License (https://creativecommons.org/licenses/by/4.0/).

_In the living larva however illuminati only occurs in the neural stem cells. Whether this observation reflects plasticity in the genetic program of this tissue to generate multiple cell fates is highly speculative and is way beyond the scope of this article

This is totally correct. The likely involvement of genetic programme plasticity in illuminati remains speculative. In this regard, the new data added in the revised version showing that illuminati is much less frequent (to say the least; may be inexistent) in the somatic and germline SCs of testis and ovaries strongly suggests that it may not be a general feature of SCs.

Some considerations for the future:

__I assume that all the components of the bipartite system are in heterozygous conditions in the analyzed larval brains. Otherwise my following comments do not make sense. The trigger leading to inactivation of the reporter gene could be direct, by aiming at the Tub-GAL80 transgene, or could be an indirect effect due to the inactivation of the neighboring chromatin. In the 1st case this would lead to heterozygocity, but in the 2nd case both homologs could be inactivated. I am wondering if the authors have thought of probing for the activity of the genes immediately adjacent to the integration platform that carry their Tub-GAL80. They could test by in situ hybridization to see if the neighboring gene(s) also become inactive in the Illuminati clones. Furthermore, it would be instructive to know the status of the white+ gene adjacent to the Tub-GAL80 transgene in the illuminati clones. . Finally, what happen in case when the Tub-GAL80 transgene is in homozygous conditions?

The reviewer's assumption is correct: all the components of the bipartite system are in heterozygous condition in most of the analysed larval brains. And the reviewers' proposal to investigate whether Illuminati may extend beyond the Gal80 gene and affect neighbouring genes is an excellent idea.

We have not carried out the suggested FISH experiments, but we do have solid data regarding the possible effect of illuminati upon the homologue chromosome. One example is line X, 5B8 19E7. As shown in Figure 4C, X,5B8 19E7 / X heterozygous brains present GFP cells at a rate of nearly 8 GFP+ clones per brain. In contrast, in X,5B8 19E7/ X,5B8 19E7 homozygous larvae only a third of the brains are GFP+ and the frequency of illuminati clones per brain lobe drops to 0.5. If inhibition of a given Gal80 also inhibited the corresponding Gal80 in the homologue chromosome, no drop in illuminati frequency should be expected.

Minor comments

__I am confused by the experiments reported in Supplementary Figure3, as I would expect that sas4 minus clones would fail to divide, unless there is perdurance of the SAS4 protein. Please explain.

There is extensive evidence showing that rather counterintuitively, in many species, centrioles are not essential for cell division (Basto et al., 2006; Lambrus et al, 2015; Meitinger et al 2020). In normal mammalian cells, a mitotic surveillance pathway arrests the cell cycle upon centrosome loss, but inactivation of this pathway allows acentriolar cells to divide (reviewed in Lambrus and Holland, 2017. In Drosophila, which lacks this pathway, cells can progress through mitosis without centrioles (Basto et al, 2006).

_Could have used the Bischof et al 102D line on the 4th.

Thanks for the suggestion. Indeed, our large collection of Gal80 insertions is still lacking one in the 4th chromosome.

© 2022. Published by The Company of Biologists under the terms of the Creative Commons Attribution License (https://creativecommons.org/licenses/by/4.0/).

Second decision letter

MS ID#: DEVELOP/2022/200808

MS TITLE: Illuminati, a novel form of gene expression plasticity in Drosophila neural stem cells

AUTHORS: Alix Goupil, Jan Peter Heinen, Riham Salame, Fabrizio Rossi, Jose Reina, Carole Pennetier, Anthony Simon, Anxela Louzao, Allison Bardin, Renata Basto, and Cayetano Gonzalez
ARTICLE TYPE: Research Article

I am happy to tell you that your manuscript has been accepted for publication in Development, pending our standard ethics checks.

© 2022. Published by The Company of Biologists under the terms of the Creative Commons Attribution License (https://creativecommons.org/licenses/by/4.0/).
